# Bioactive extracellular vesicles from a subset of endothelial progenitor cells rescue retinal ischemia and neurodegeneration

**DOI:** 10.1172/jci.insight.155928

**Published:** 2022-06-22

**Authors:** Kyle V. Marra, Edith Aguilar, Guoqin Wei, Ayumi Usui-Ouchi, Yoichiro Ideguchi, Susumu Sakimoto, Martin Friedlander

**Affiliations:** 1 Department of Molecular Medicine, The Scripps Research Institute, La Jolla, California, USA.; 2 Department of Bioengineering, University of California, San Diego, La Jolla, California, USA.; 3 Department of Ophthalmology, Osaka University Graduate School of Medicine, Osaka, Japan.; 4 Lowy Medical Research Institute, La Jolla, California, USA.

**Keywords:** Ophthalmology, Vascular Biology, Adult stem cells, Neurodegeneration, Retinopathy

## Abstract

Disruption of the neurovascular unit (NVU) underlies the pathophysiology of various CNS diseases. One strategy to repair NVU dysfunction uses stem/progenitor cells to provide trophic support to the NVU’s functionally coupled and interdependent vasculature and surrounding CNS parenchyma. A subset of endothelial progenitor cells, endothelial colony-forming cells (ECFCs) with high expression of the CD44 hyaluronan receptor (CD44^hi^), provides such neurovasculotrophic support via a paracrine mechanism. Here, we report that bioactive extracellular vesicles from CD44^hi^ ECFCs (EVs^hi^) are paracrine mediators, recapitulating the effects of intact cell therapy in murine models of ischemic/neurodegenerative retinopathy; vesicles from ECFCs with low expression levels of CD44 (EVs^lo^) were ineffective. Small RNA sequencing comparing the microRNA cargo from EVs^hi^ and EVs^lo^ identified candidate microRNAs that contribute to these effects. EVs^hi^ may be used to repair NVU dysfunction through multiple mechanisms to stabilize hypoxic vasculature, promote vascular growth, and support neural cells.

## Introduction

The neurovascular unit (NVU) consists of neurons, vascular endothelial cells, extracellular matrix, and perivascular astrocytes, microglia, and pericytes, together functioning to maintain the blood-brain/retinal barrier and local CNS homeostasis. Disruption of the NVU is central to the pathophysiology of various ischemic/neurodegenerative diseases of the CNS, including ischemic stroke, Parkinson’s, Alzheimer’s, multiple sclerosis, amyotrophic lateral sclerosis, and diabetic retinopathy (1–3). Ischemia promotes CNS remodeling, where neurovascular crosstalk between the neurons, glia, and microvascular cells of the NVU supports a microenvironment that favors tissue recovery. Since multicellular crosstalk between local vascular networks and the neurons they supply in the NVU is critical to maintaining physiological function, one regenerative therapeutic strategy is to repair the dysfunctional NVU using progenitor and/or stem cells to provide support to the complex of vascular endothelial cells and surrounding CNS parenchyma that are functionally coupled and interdependent (4).

Recent studies support the use of the endothelial progenitor cells known as endothelial colony-forming cells (ECFCs) to achieve this effect. ECFCs home to areas of ischemia and exhibit potent rescue effects in numerous animal models of ischemic/neurodegenerative CNS diseases (5–10). As a readily accessible and visualized extension of the brain, the retina is an exceptional experimental system for modeling ischemic/neurodegenerative CNS diseases for the preclinical development of novel therapeutics. Experiments in murine models of retinal ischemia/degeneration have provided proof-of-concept evidence that neurovasculotrophic support by ECFCs (and other stem/progenitor cells) protects retinal neurons from undergoing apoptosis (11–17). Evidence has suggested that the therapeutic mechanism of ECFCs is predominantly paracrine. Despite their potent rescue effects in ischemic/neurodegenerative CNS disease models in vivo, low levels of ECFC engraftment within cerebral vasculature have been observed (5–10). ECFCs home to ischemic areas and assume perivascular positions within the retina (11–13), and injection of ECFC-conditioned media (CM) in mouse models of ischemic retinopathy recapitulate the rescue effects observed following cell therapy (12).

Extracellular vesicles from ECFCs are a promising new addition to the armamentarium of paracrine mediators that confer therapeutic benefit by transferring bioactive cargo. Within the NVU, extracellular vesicles (EVs) are bidirectional messengers between the brain and periphery as well as within the brain (18). EVs from ECFCs activate an angiogenic program in target endothelial cells by horizontally transferring RNA (19). The majority of intravesicular microRNA (miR) in EVs from ECFCs target angiogenic functional categories, including vascular development and cell migration (20).

Previous work suggests only a bioactive subset of ECFCs have functional trophic effects in vivo. While ECFCs with high expression of hyaluronan receptor CD44 (CD44hi) rescue animal models of retinal ischemia (oxygen-induced retinopathy, OIR) and neurodegeneration (Pde6b^rd10/rd10^ RD10 mice) via a paracrine mechanism, ECFCs with low CD44 expression (CD44^lo^) have little effect (12). The current study exploited these findings to identify trophic miRs in shed EVs from bioactive CD44^hi^ ECFCs (EVs^hi^). EVs^hi^ recapitulated the neurovasculotrophic rescue effects of intact cell transplantation in OIR mice and RD10 mice, a model of inherited retinal degeneration; EVs^lo^ had little effect. In OIR mice, EVs^hi^ homed to sites of ischemia and neovascularization and incorporated within neovasculature and perivascular microglia/macrophages. Reduction of intravesicular miR via lentiviral DICER1 knockdown (KD) attenuated the effects of CD44^hi^ ECFCs and EVs^hi^ in OIR mice. Small RNA sequencing identified miRs that were upregulated in EVs^hi^ relative to EVs^lo^ and trophic in OIR mice. Individual KD of miR-23a-3p or miR-503-5p or combinatorial miR KD attenuated the effects of EVs^hi^. Overall, EVs^hi^ rely upon intravesicular neurovasculotrophic miRs and target the NVU in ischemic/neurodegenerative diseases of the CNS to stabilize retinal vasculature to hypoxia, stimulate vascular growth, and provide trophic support to neurons.

## Results

### Culture, immunophenotype, sorting, and transfection of ECFCs.

**Figure 1:**
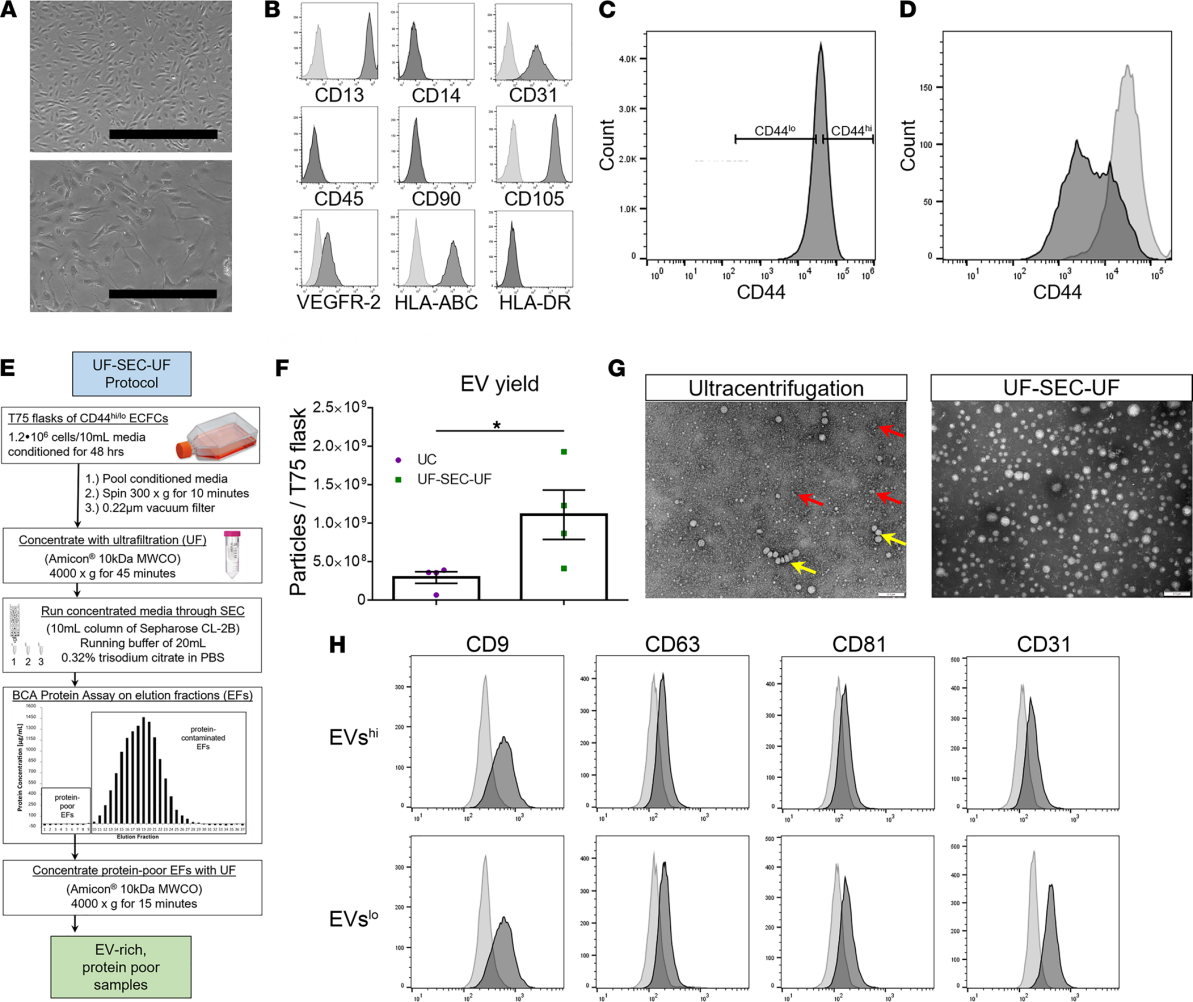
ECFC culture and EV isolation. (**A**–**D**) ECFC characterization and CD44 sorting and KD. (**A**) Representative images of confluent ECFC colonies taken at 5× (top) and 10× (bottom) original magnification. Scale bar: 500 μm (top), 200 μm (bottom). (**B**) Immunophenotypic characterization of ECFCs. Representative flow cytometry histograms of ECFCs demonstrated positive expression of CD13, CD31, CD105, and HLA-ABC and negative expression of hematopoietic markers CD14 and CD45, mesenchymal stem cell marker CD90, as well as HLA-DR (right-shifted, black-filled curves in comparison with gray-filled curves representing the appropriate isotype controls; *n* = 3 replicates). (**C**) Representative gating strategy to sort CD44^hi^ and CD44^lo^ ECFCs using FACS. (**D**) Flow cytometric analysis of CD44 in ECFCs-shCD44 (black-filled curves) and ECFCs-scrRNA (gray-filled curves) following lentivirus-mediated transduction of ECFCs with shCD44. (**E**–**H**) EV isolation protocol, yield, morphology, and immunophenotype. (**E**) Schematic of UF-SEC-UF protocol. (**F**) UF-SEC-UF obtained a significantly higher EV yield than differential UC. Two-tailed Student’s *t* test; *n* = 4 EV isolations. Error bars represent SEM. (**G**) TEM of EV samples isolated via differential UC (left) or UF-SEC-UF (right). Differential UC samples demonstrated aggregation of macromolecules (red arrows) and EVs (yellow arrow). UF-SEC-UF produced EV samples devoid of contaminating aggregates. Scale bars: 0.2 μm. (**H**) Representative magnetic bead–assisted flow cytometry histograms of EVs^hi^ and EVs^lo^. Both populations positively expressed tetraspanins CD9, CD63, and CD81, as well as endothelial marker CD31 (right-shifted, black filled curves compared with gray-filled curves of negative control samples; *n* = 3 replicates). *P < 0.05. MWCO, molecular weight cutoff.

Human umbilical cord blood–derived (UCB-derived) ECFCs exhibited characteristic cobblestone morphology (Figure 1A) (12). The ECFC immunophenotype was confirmed via flow cytometric analysis demonstrating positive expression of CD13, CD31, CD105, VEGFR-2, and HLA-ABC and negative expression of hematopoietic markers CD14 and CD45, mesenchymal marker CD90, and HLA-DR as previously reported (Figure 1B) (12). ECFCs were sorted into CD44^hi^ and CD44^lo^ populations (Figure 1C). KD of CD44 expression in ECFCs was achieved using lentiviral transfection with short hairpin RNA to CD44 (ECFCs-shCD44), and control ECFCs were transfected with scramble RNA (ECFCs-scrRNA) with an efficiency of 90.1% and 93.1%, respectively, measured using confocal microscopy. CD44 KD efficiency was 63.8% on real-time quantitative PCR (RT-qPCR), and KD was validated by flow cytometry (Figure 1D). ECFCs were lentivirally transfected with short hairpin RNA to DICER1 (ECFCs-shDICER1), and another control line of ECFCs-scrRNA was generated. ECFCs-shDICER1 KD efficiency was 63.8% by RT-qPCR.

### Isolation and characterization of EVs.

An EV isolation protocol was designed to optimize yield and purity of EVs harvested from CM (21). Serial ultrafiltration (UF), size-exclusion chromatography (SEC), and then repeat UF (UF-SEC-UF) produced soluble protein–poor, EV-enriched samples. Early SEC elution fractions (EFs) contained undetectable levels of soluble protein in a BCA protein assay (Figure 1E) and contained EVs expressing exosomal marker CD63 on magnetic bead–assisted flow cytometry. Early protein-poor, EV-rich EFs were concentrated by UF to produce UF-SEC-UF samples, which were higher in EV yield than samples isolated by differential ultracentrifugation (Figure 1F). On transmission electron microscopy (TEM), EVs isolated via differential ultracentrifugation (UC) demonstrated characteristic vesicle size/morphology but contained macromolecular and vesicular aggregates. Characteristic EV size/morphology was also observed in UF-SEC-UF samples, but macromolecular and vesicular aggregates were not observed (Figure 1G). Nanoparticle tracking analysis corroborated the appropriate EV size distribution.

No differences between the immunophenotypes of EVs^hi^ versus EVs^lo^ were observed on magnetic bead–assisted flow cytometry. EVs^hi^ and EVs^lo^ expressed exosomal tetraspanins CD9, CD63, and CD81 and endothelial marker CD31 on magnetic bead–assisted flow cytometry (Figure 1H).

### EVs^hi^ rescue the OIR mouse model of ischemic retinopathy.

**Figure 2:**
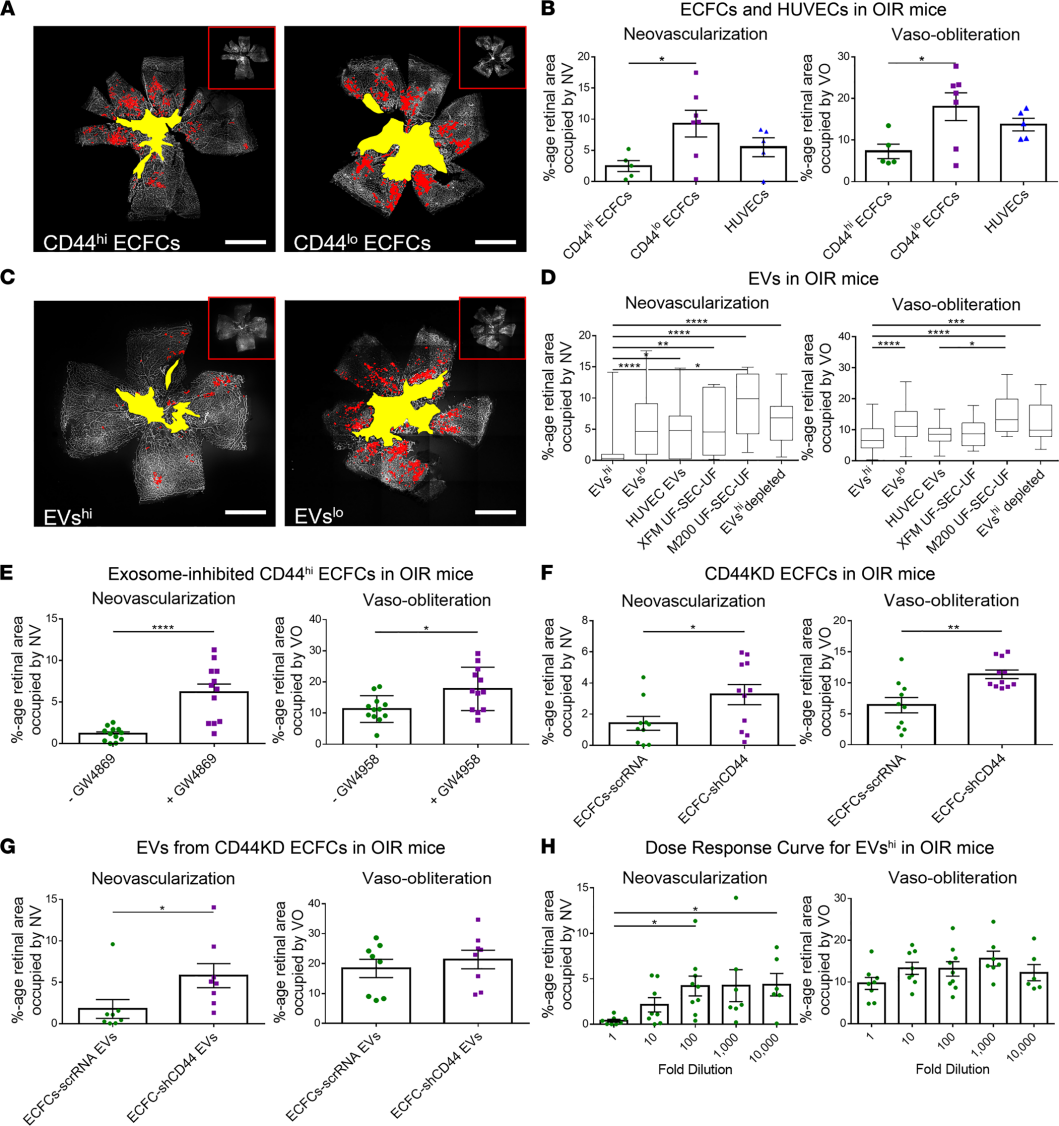
ECFCs with high CD44 expression and their shed EVs rescue OIR mice. (**A** and **B**) CD44^hi^ ECFCs rescued OIR mice. (**A**) Representative images and (**B**) quantification of NV (red) and VO (yellow) of retinal flat mounts from OIR mice. One-way ANOVA with Tukey’s; *n* = 7 retinas for CD44^hi^ ECFCs, *n* = 7 retinas for CD44^lo^ ECFCs, *n* = 5 retinas for HUVECs. (**C** and **D**) EVs^hi^ rescued OIR mice. (**C**) Representative images and (**D**) quantification of retinal flat mounts. Inserts in **A** and **C** depict the original unquantified images; scale bars: 1 mm. Additional controls included EVs^lo^, HUVEC EVs, nonconditioned ECFC and HUVEC media subjected to UF-SEC-UF (XFM UF-SEC-UF and M200 UF-SEC-UF, respectively), and EVs^hi^ sample depleted of vesicles via overnight UC (EVs^hi^ depleted). Data in **D** are represented as a box-and-whisker plot where the top and bottom of the box represent mean of the upper and lower quartiles, horizontal line within the box represents the mean, and bars outside the box represent the min and max data points. One-way ANOVA with Tukey’s; *n* = 100 retinas for EVs^hi^, *n* = 102 retinas for EVs^lo^, *n* = 10 retinas for HUVEC EVs, *n* = 11 retinas for XFM UF-SEC-UF, *n* = 11 retinas for M200 UF-SEC-UF, *n* = 12 retinas for EVs^hi^ depleted. (**E**) Pharmacologic exosome inhibition of CD44^hi^ ECFCs via GW4869 (20 μM) in DMSO (+GW4869, *n* = 12 retinas) attenuated the effects of CD44^hi^ ECFCs compared with cells incubated with DMSO alone (-GW4869, *n* = 12 retinas). Two-tailed Student’s *t* test. (**F** and **G**) ECFCs-shCD44 and their EVs failed to rescue OIR mice. Quantification of NV and VO in mice injected with (**F**) ECFCs-scrRNA (*n* = 10 retinas) versus ECFCs-shCD44 (*n* = 11 retinas) and (**G**) EVs from ECFCs-scrRNA (*n* = 10 retinas) versus EVs from ECFCs-shCD44 (*n* = 11 retinas). Two-tailed Student’s *t* test. (**H**) Dose-response curve of OIR mice injected with EVs^hi^. Mice were treated with a starting dose of 1.25 × 10^6^ particles/0.5 μL/eye and serial 10-fold dilutions. Kruskal-Wallis test with Dunn’s multiple-comparison test; *n* = 6–9 retinas per group. Error bars represent SEM. *P < 0.05, **P < 0.01, ***P < 0.001, ****P < 0.0001.

Intravitreal injections of CD44^hi^ ECFCs on postnatal day 12 (P12) more effectively rescued the neovascularization (NV) and vaso-obliteration (VO) on P17 in OIR mice than CD44^lo^ ECFCs or PBS control, as previously reported (12). Injections of human umbilical vein endothelial cells (HUVECs) failed to rescue OIR mice (Figure 2, A and B). EVs^hi^ rescued NV and VO in OIR mice while EVs^lo^ or PBS vehicle did not. HUVEC EVs also did not rescue OIR mice, suggesting these rescue effects were endothelial progenitor cell specific. To control for components of cell culture media that may remain in UF-SEC-UF samples, nonconditioned ECFC media (xeno free media [XFM] UF-SEC-UF) and vesicle-depleted HUVEC media (M200 UF-SEC-UF) were subjected to UF-SEC-UF and injected into OIR mice but had no effect. EVs^hi^ sample depleted of vesicles by UC at 120,000*g* for 18 hours failed to rescue OIR (Figure 2, C and D), further suggesting rescue effects were attributable to vesicles within the EVs^hi^ sample. CD44^hi^ ECFCs treated with neutral sphingomyelinase inhibitor GW4869, the most widely used pharmacological inhibitor of exosome biogenesis and release, no longer rescued OIR mice (Figure 2E) (22–24).

The capacity to which CD44 expression on ECFCs correlates with neurovascular benefit was investigated by injecting ECFCs-shCD44 and their EVs into OIR mice. ECFCs-shCD44 failed to reduce NV and VO whereas ECFCs-scrRNA rescued OIR mice (Figure 2F). EVs from ECFCs-shCD44 failed to rescue NV when compared to eyes treated with ECFCs-scrRNA EVs (Figure 2G).

EVs from all 4 UCB donors rescued OIR mice, suggesting that therapy may be derived from any healthy donor or banks of pooled UCB. While most EVs were prepared fresh before injection, samples frozen at –80°C for as long as 1 year retained therapeutic function in OIR mice, corroborating studies suggesting the integrity and function of ECFC-derived EVs are stable in long-term storage with multiple freeze-thaw cycles (25, 26). To determine treatment potency, a dose-response experiment injecting EVs^hi^ with serial 10-fold dilutions from an initial dose of 1.25 × 10^6^ particles/eye was performed. The effects of EVs^hi^ on NV were significantly reduced following 100-fold dilution (Figure 2H).

Experiments described thus far were performed with injections of vesicles on P12 (immediately upon return from hyperoxia to room air), the time point at which the ischemic drive is initiated. To investigate the impact of treatment timing on efficacy, EVs^hi^ were injected into OIR mice immediately before entering hyperoxia (on P7) or 2 days after pups returned to normoxia (on P14). Injection with EVs^hi^ on P7 rescued the OIR phenotype compared with injections with EVs^lo^ (Supplemental Figure 1A; supplemental material available online with this article; https://doi.org/10.1172/jci.insight.155928DS1). EVs^hi^ failed to rescue OIR mice when injected on P14 (Supplemental Figure 1B). The rescue of OIR mice following P7 injections informed our hypothesis that EVs^hi^ treatment can promote physiological vascular growth during VO. Injections were performed at P7 and retinas were evaluated at time points within (P10 and P12) and near the end (P14) of the vaso-obliterative phase of OIR. Decreased VO following EVs^hi^ injection suggested that EVs^hi^ promoted vascular growth during the vaso-obliterative phase of OIR (Supplemental Figure 2A).

Retinal vascularization is typically completed at birth in humans, but the retinas of infants born prematurely may be incompletely vascularized, thus carrying increased risk of retinopathy of prematurity (ROP) (27). The murine OIR model mimics the hallmark features of human ROP — initial VO and subsequent NV — as well as other complications, including vascular leakage (28). Following the observation that EVs^hi^ rescued OIR mice, we investigated the effects of EVs^hi^ treatment on vascular development. These experiments may aid our understanding of whether EVs^hi^ can prevent neovascularization while allowing normal vascularization in premature infants at risk of ROP. Newborn mice are a useful developmental model of retinal vasculature since the vascularization of the murine retina occurs postnatally. Just before birth, the retinal vasculature begins developing at the optic nerve head in mice, extending radially until reaching the peripheral retina by P28 (16). Wild-type pups were treated on P2 with EVs^hi^, EVs^lo^, or PBS vehicle or were untreated. Mice were sacrificed on P5 and the percentage of the retina covered with vasculature was quantified. No effect on retinal vasculature was observed following treatment with EVs^hi^ or EVs^lo^, suggesting EV treatment does not disturb normal retinal vascular development (Supplemental Figure 2, B and C).

Although OIR mice classically model retinal ischemic vasculopathy, these mice also exhibit some neurodegeneration, evidenced by thinning of the inner nuclear layer (INL) and inner plexiform layer (IPL). EVs^hi^ were injected on P12 and the thickness of the INL and IPL were measured on P30 to determine if EVs^hi^ exhibit neuroprotection in OIR mice. EVs^hi^ restored the INL and IPL thickness to 92% and 93% of that observed in normoxic mice, respectively; no such significant effect was observed following treatment with EVs^lo^ (Supplemental Figure 3A). In agreement with published studies, outer nuclear layer (ONL) thickness was not affected by OIR (12).

To assess treatment effects on neural function, electroretinography (ERG) was performed on P30 following P12 injection with EVs^hi^ versus PBS vehicle. Following injection of EVs^hi^, the scotopic B wave amplitude was unchanged, the photopic B wave amplitude was significantly improved, and the flicker response trended toward improvement but was not statistically significant (Supplemental Figure 3B). These data demonstrated that EVs^hi^ recapitulated the published effects of CD44^hi^ ECFCs on improving inner retinal thickness and function in OIR mice (12).

### EVs^hi^ home to areas of retinal ischemia and associate with preretinal neovascular tufts and perivascular microglia and macrophages in OIR mice.

**Figure 3:**
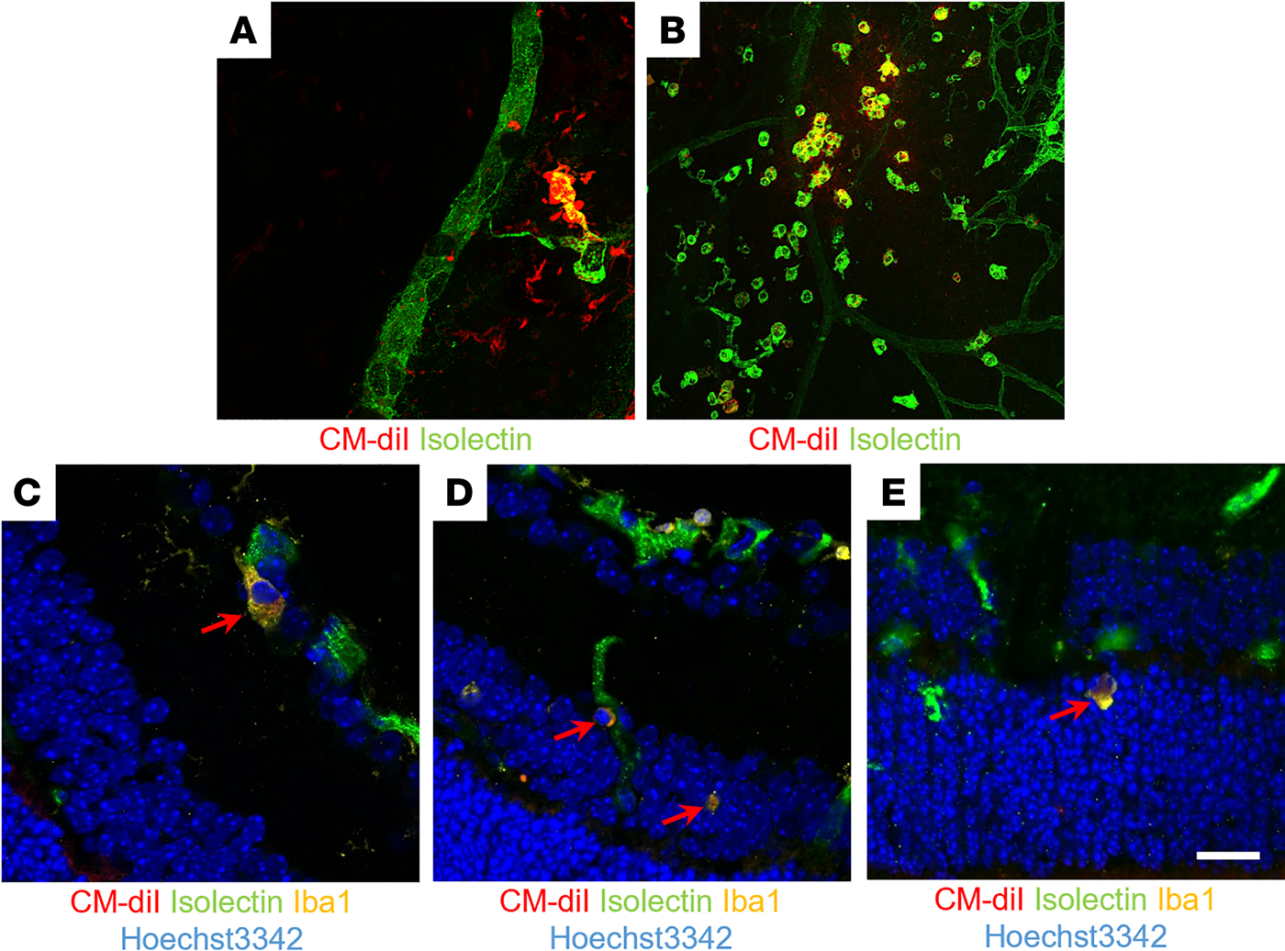
EVs^hi^ home to areas of retinal ischemia and associate with perivascular macrophages/microglia in OIR mice. CM-DiI–labeled EVs (red) were intravitreally injected into OIR mice on P12 and localized with immunohistochemistry. (**A** and **B**) Flat mounts of retinas harvested on P17 showed colocalization of EVs^hi^ within neovascularization in **A** and superficial macrophages/microglia in ischemic regions in **B**. (**C**–**E**) Cross sections of retinas harvested on P15 demonstrated accumulation of CM-DiI–labeled EVs^hi^ (red) within perivascular Iba1^+^ microglia/macrophages (yellow) with Hoechst 3342 nuclear staining (blue) located in the retinal ganglion cell layer in **C**, the inner nuclear layer in **D**, and the outer nuclear layer in **E**. Red arrows indicate colocalization of EVs^hi^ and microglia. Scale bar: 20 μm.

EVs^hi^ stained with lipophilic CM-DiI were intravitreally injected into OIR mice on P12. Retinas were harvested at 2, 4, 6, and 12 hours after injection and on P13, P14, P15, and P17. Immunohistochemistry of retinal flat mounts harvested as early as 2 hours after injection and as late as P17 showed CM-DiI–labeled EVs^hi^ within preretinal neovascular tufts (Figure 3A) and superficial macrophages/microglia (Figure 3B). Immunohistochemistry of retinal cross sections demonstrated the colocalization of CM-DiI labeled EVs^hi^ with perivascular ionized calcium binding adaptor molecule 1–positve (Iba1^+^) macrophages/microglia in the retinal ganglion cell layer, the INL, and the ONL (Figure 3, C–E).

### EVs^hi^ demonstrated neurovasculotrophic effects in mice with inherited retinal degeneration.

The neurovasculotrophic effects of EVs^hi^ were rigorously evaluated in RD10 mice, a model of inherited retinal degeneration that exhibits both vascular and neural defects. The RD10 model contains a missense mutation in the catalytic PDE6 β subunit typically responsible for hydrolyzing cyclic GMP in photoreceptors’ response to light. As a result, photoreceptor cell death begins concurrently with atrophy of the deep vascular plexus on P21. Between P20 and P25, cells in the ONL die primarily by apoptosis, resulting in an ONL that degenerates to 3 to 5 cell layers thick. By P45, the ONL is 1 cell layer thick (17, 29, 30). RD10 mice were treated on P14 and a number of outcome measurements were quantified. These included the degeneration of the deep and intermediate vascular plexus, ONL apoptosis and thickness, neural function on ERG, and photoreceptor cell density.

**Figure 4:**
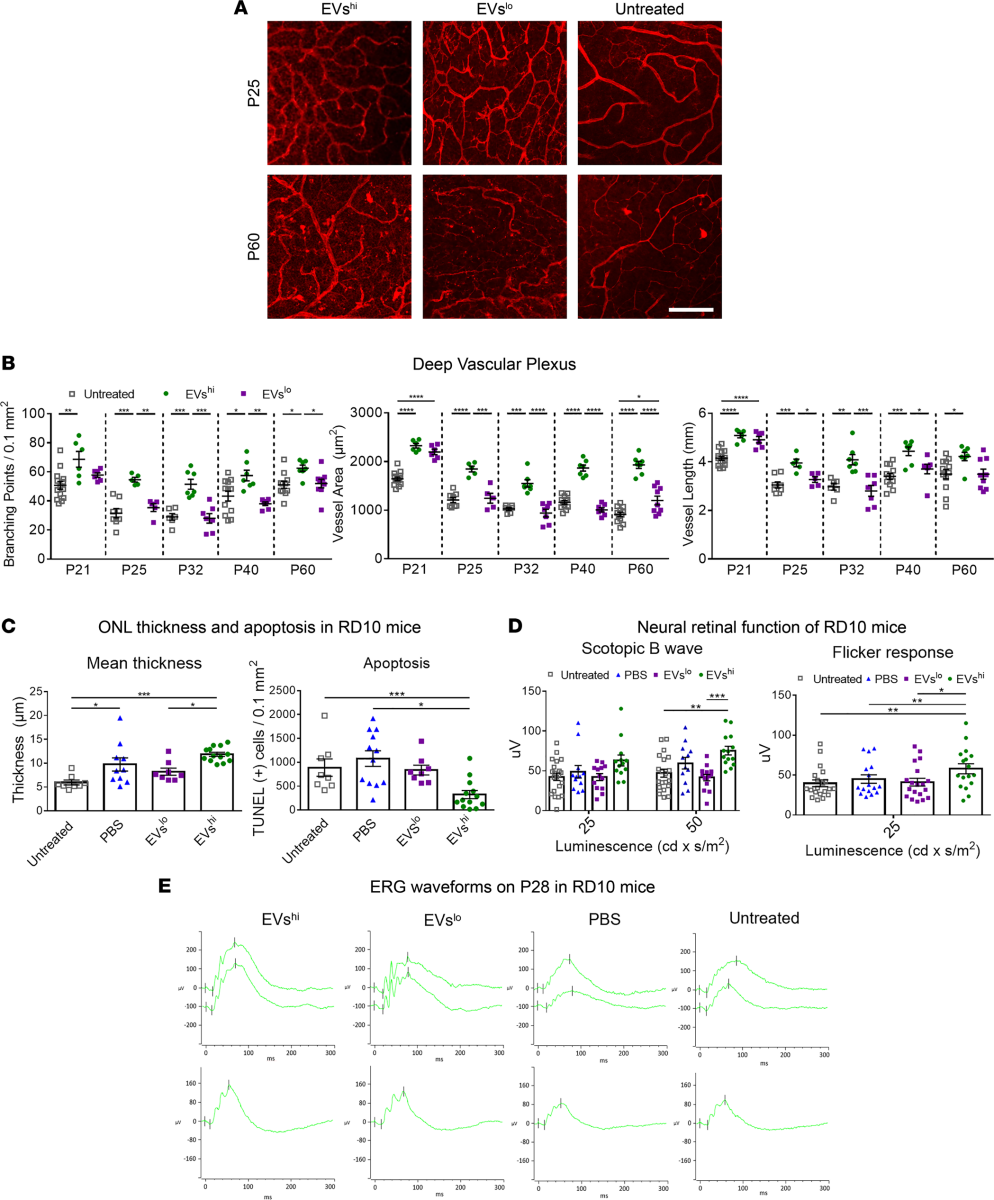
EVs^hi^ provide neurovasculotrophic support to inherited retinal degeneration mice. (**A**) Representative images of the deep vascular plexus in Isolectin *Griffonia simplicifolia*-IB4–stained (GS-IB4–stained) flat-mounted P25 and P60 retinas of RD10 mice treated on P14 with either EVs^hi^ or EVs^lo^ or untreated mice. Scale bar: 100 μm. (**B**) Treatment of RD10 mice with EVs^hi^ delayed vascular atrophy. Quantification of the branching points (left), total vessel area (middle), and total vessel length (right) in the deep vascular plexus at P21, P25, P32, P40, and P60 demonstrated EVs^hi^ delayed atrophy of the deep vascular plexus. One-way ANOVA with Tukey’s analysis; *n* = 5–9 retinas in EV groups, *n* = 8–14 retinas in untreated groups. Error bars represent SEM. (**C**) Immunohistochemistry of retinal cross sections harvested on P28 from RD10 mice treated P14 demonstrated a neuroprotective role of EVs^hi^. Quantification of the ONL thickness (left) and density of apoptosis in the ONL via TUNEL staining (right). One-way ANOVA with Tukey’s analysis; *n* = 13 retinas for EVs^hi^, *n* = 9 retinas for EVs^lo^, *n* = 10 retinas for PBS, *n* = 8 retinas for untreated. (**D**) EVs^hi^ promoted functional rescue of the neural retina in RD10 mice. ERG measurements on P42 showed pronounced and lasting improvement in both dark-adapted (rod-driven scotopic B wave, left) and light-adapted (cone-driven flicker response, right) retinal function following P14 treatment with EVs^hi^. One-way ANOVA with Tukey’s analysis; *n* = 12–14 retinas for EVs^hi^, *n* = 12–14 retinas for EVs^lo^, *n* = 12 retinas for PBS, *n* = 20–22 retinas for untreated. Error bars in all figures represent SEM. (**E**) Representative waveforms of the mean ERG readings on P28 of RD10 mice treated on P14 with EVs^hi^, EVs^lo^, or PBS or untreated. *P < 0.05, **P < 0.01, ***P < 0.001, ****P < 0.0001.

*Z*-stack images of retinal flat mounts were used to investigate the effects of EVs^hi^ on the delay of vascular atrophy in RD10 mice. Vessels in the deep vascular plexus demonstrated increased branching points, total area, and total length at multiple time points (P21, P25, P32, P40, and P60) following P14 injection with EVs^hi^ in comparison with eyes treated with EVs^lo^, PBS, or untreated controls (Figure 4, A and B). Atrophy of the intermediate plexus at these time points was also attenuated following treatment with EVs^hi^ (Supplemental Figure 4).

To evaluate neuroprotection in RD10 mice, ONL thickness and apoptosis as well as neural function were investigated following P14 treatment. ONL thickness at P21 (Supplemental Figure 5A) and at P28 (Figure 4C) was more well preserved, and apoptosis was reduced in the ONL of retinas treated with EVs^hi^. Neural retinal function was measured by full-field ERG. EVs^hi^ improved both scotopic and photopic signals at P42 compared with eyes injected with EVs^lo^ or PBS, or untreated controls (Figure 4D). In a second experiment, we assessed whether multiple treatments with EVs^hi^ may further augment retinal function. RD10 mice were injected once (at P14) or twice (at P14 and P21), and ERG responses were measured at P28. Single injection of EVs^hi^ rescued the scotopic B wave amplitude, photopic B wave amplitude, and flicker response. Repeated injection of EVs^hi^ significantly improved scotopic and photopic B wave amplitudes relative to PBS controls. However, ERG recordings were decreased in mice injected twice when compared to mice receiving single injections in the corresponding treatment group (Supplemental Figure 5, B and C). Representative waveforms of RD10 mice treated on P14 and sacrificed at P28 are provided in Figure 4E. These data suggested that repeated intravitreal injections may have deleterious effects, which has been observed in mice and humans (31, 32). EVs from ECFCs-shCD44 and ECFCs-scrRNA were injected on P14 and ERG recordings were measured on P28. Treatment with EVs from ECFCs-scrRNA significantly increased scotopic B wave amplitude and trended toward improving photopic B wave amplitude and flicker response, but the latter effects were not statistically significant (Supplemental Figure 6). These findings supported a correlation between CD44 expression on ECFCs and the neuroprotective effects of shed EVs.

**Figure 5:**
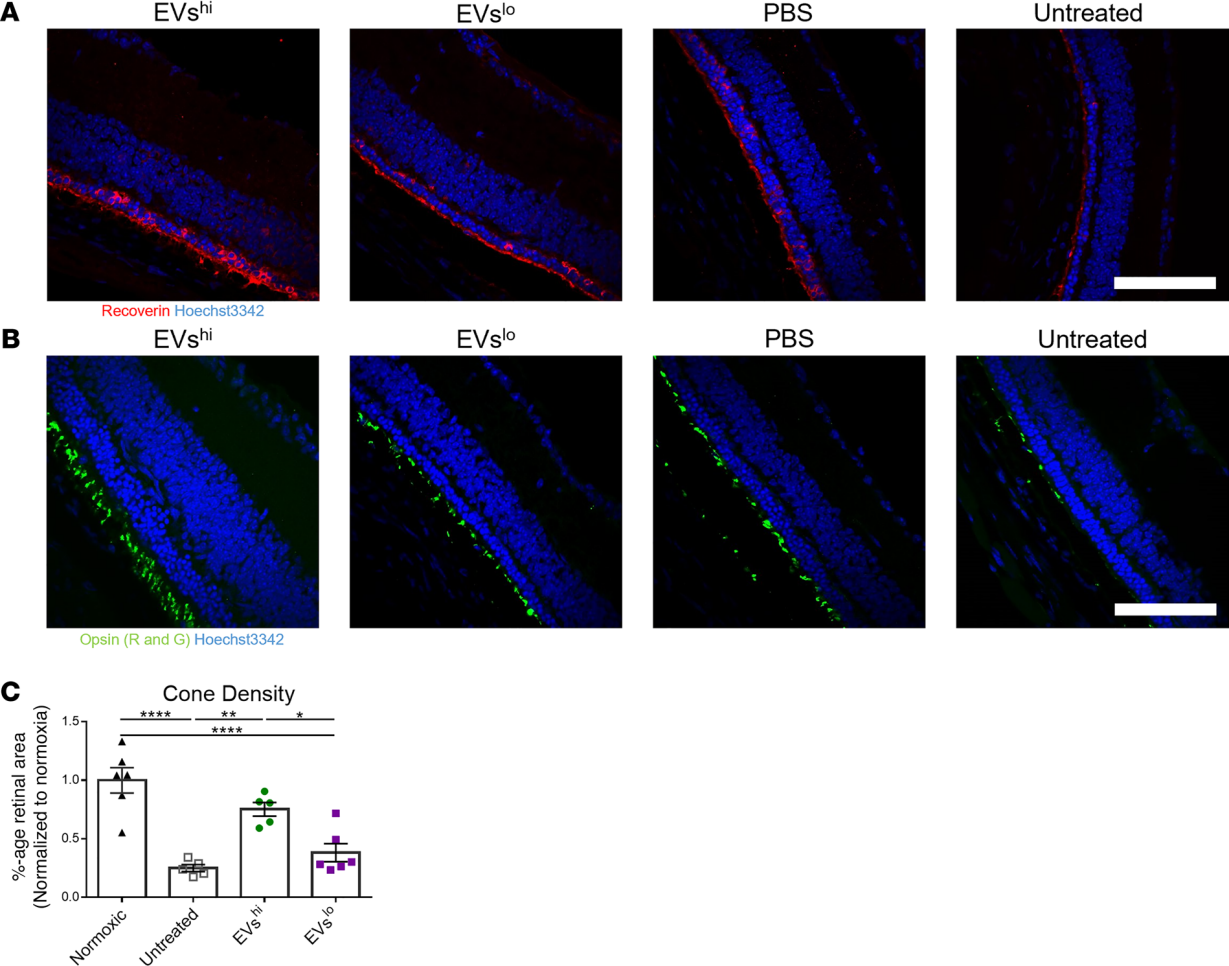
EVs^hi^ improves photoreceptor density in inherited retinal degeneration mice. (**A** and **B**) Immunohistochemistry for cone- and rod-specific markers on RD10 retinas treated P14 and harvested P28 demonstrated photoreceptor preservation by EVs^hi^. Retinal cross sections were stained for recoverin (in red) in **A** and for opsin red/green (in green) in **B** with Hoechst 3342 nuclear staining (in blue). (**C**) Quantification of cone photoreceptor density. One-way ANOVA with Tukey’s analysis; *n* = 6 eyes for each group. Scale bars: 50 μm. *P < 0.05, **P < 0.01, ****P < 0.0001.

Retinal cross sections stained for rod- and cone-specific markers at P28 qualitatively suggested increased photoreceptor density in RD10 retinas treated with EVs^hi^ at P14 (Figure 5, A and B). Similarly, retinal flat mounts stained for red/green opsin suggested increased cone receptor density in eyes treated with EVs^hi^ at P14 (Supplemental Figure 7). Quantification of these data demonstrated that relative to mice treated with EVs^lo^ or untreated controls, RD10 mice treated with EVs^hi^ exhibited significantly greater cone density, measuring 75.3% of that observed in flat mounts of normoxic C57BL/6 mouse retinas (Figure 5C).

### DICER1 KD reduces rescue effects of EVs^hi^ in OIR mice.

**Figure 6:**
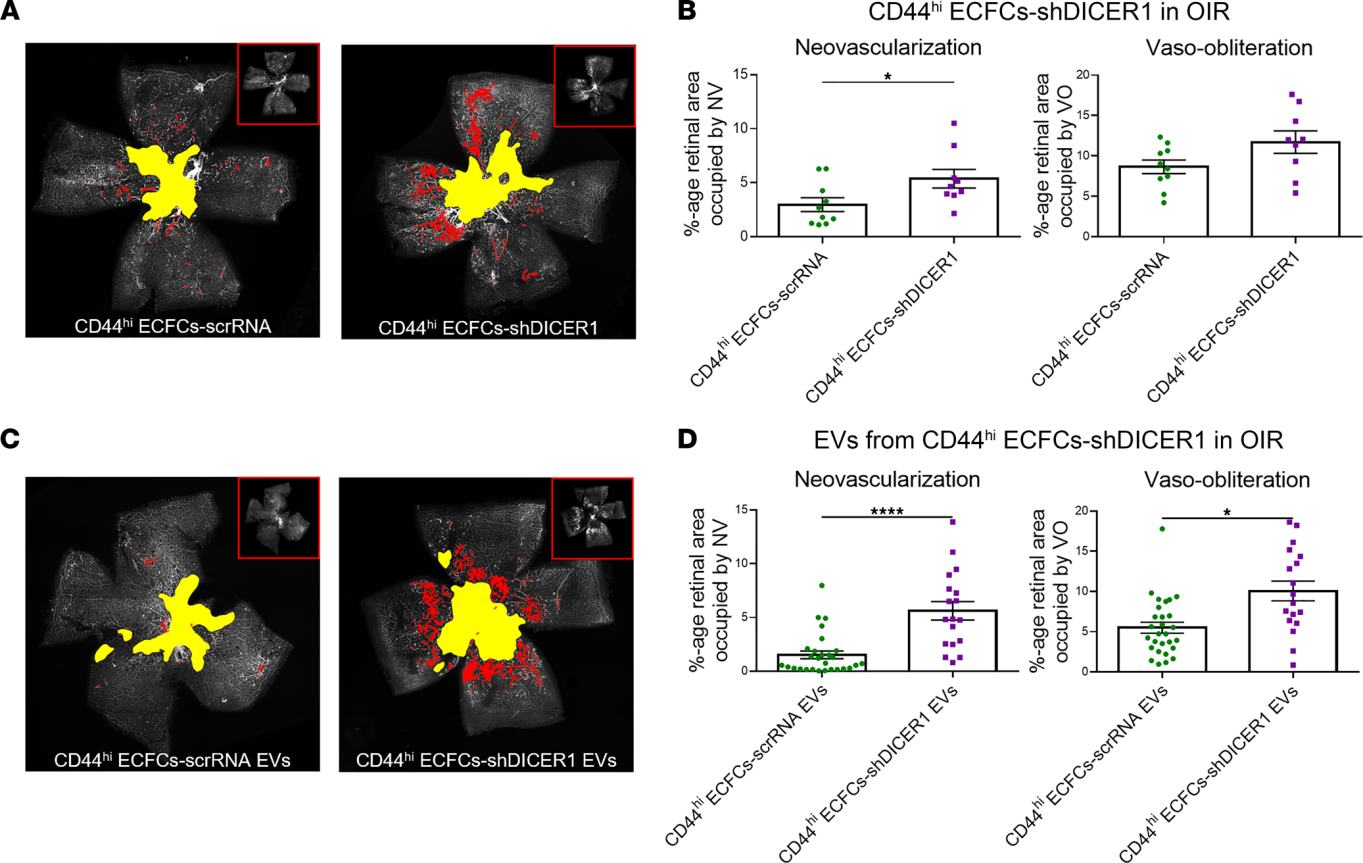
Effects of CD44^hi^ ECFCs and EVs^hi^ in OIR mice are reduced following DICER1 KD. DICER1 KD attenuated rescue effects of CD44^hi^ ECFCs in **A** and **B** and their EVs in **C** and **D** in OIR mice. (**A**) Representative images and (**B**) quantification of NV and VO in retinal flat mounts from OIR mice demonstrated that CD44^hi^ ECFCs-shDICER1 failed to rescue NV relative to mice treated with scrRNA-transfected control cells (CD44^hi^ ECFCs-scrRNA). (**C**) Representative images and (**D**) quantification of retinal flat mounts demonstrated that EVs from CD44^hi^ ECFCs-shDICER1 failed to rescue both NV and VO relative to mice treated with EVs from CD44^hi^ ECFCs-scrRNA EVs. Two-tailed Student’s *t* test; *n* = 10 retinas for CD44^hi^ ECFCs-scrRNA, *n* = 9 retinas for CD44^hi^ ECFCs-shDICER1, *n* = 28 retinas for CD44^hi^ ECFCs-scrRNA EVs, *n* = 18 retinas for CD44^hi^ ECFCs-DICER1 EVs. Error bars represent SEM. *P < 0.05, ****P < 0.0001.

Small RNA sequencing of EVs from the general population of ECFCs has demonstrated that 75% of miR reads target functional categories of vascular development or endothelial cell migration (20). EVs from ECFCs are taken up by endothelial cells in vitro and promote an angiogenic program through the horizontal transfer of miR (19, 20). We hypothesized that the superior therapeutic effects of EVs^hi^ relative to EVs^lo^ may, at least in part, be attributable to neurovasculotrophic miRs upregulated within EVs^hi^. ECFCs-shDICER1 and ECFCs-scrRNA were sorted in CD44^hi^ populations, and these cells or their EVs were intravitreally injected in separate experiments into OIR mice. In comparison with CD44^hi^ ECFCs-scrRNA, CD44^hi^ ECFCs-shDICER1 failed to rescue NV in the OIR model (Figure 6, A and B). CD44^hi^ ECFCs-shDICER1 EVs failed to reduce both NV and VO in OIR mice compared with CD44^hi^ ECFCs-scrRNA EVs (Figure 6, C and D).

### Small RNA sequencing identified candidate microRNAs within EVs^hi^.

**Figure 7:**
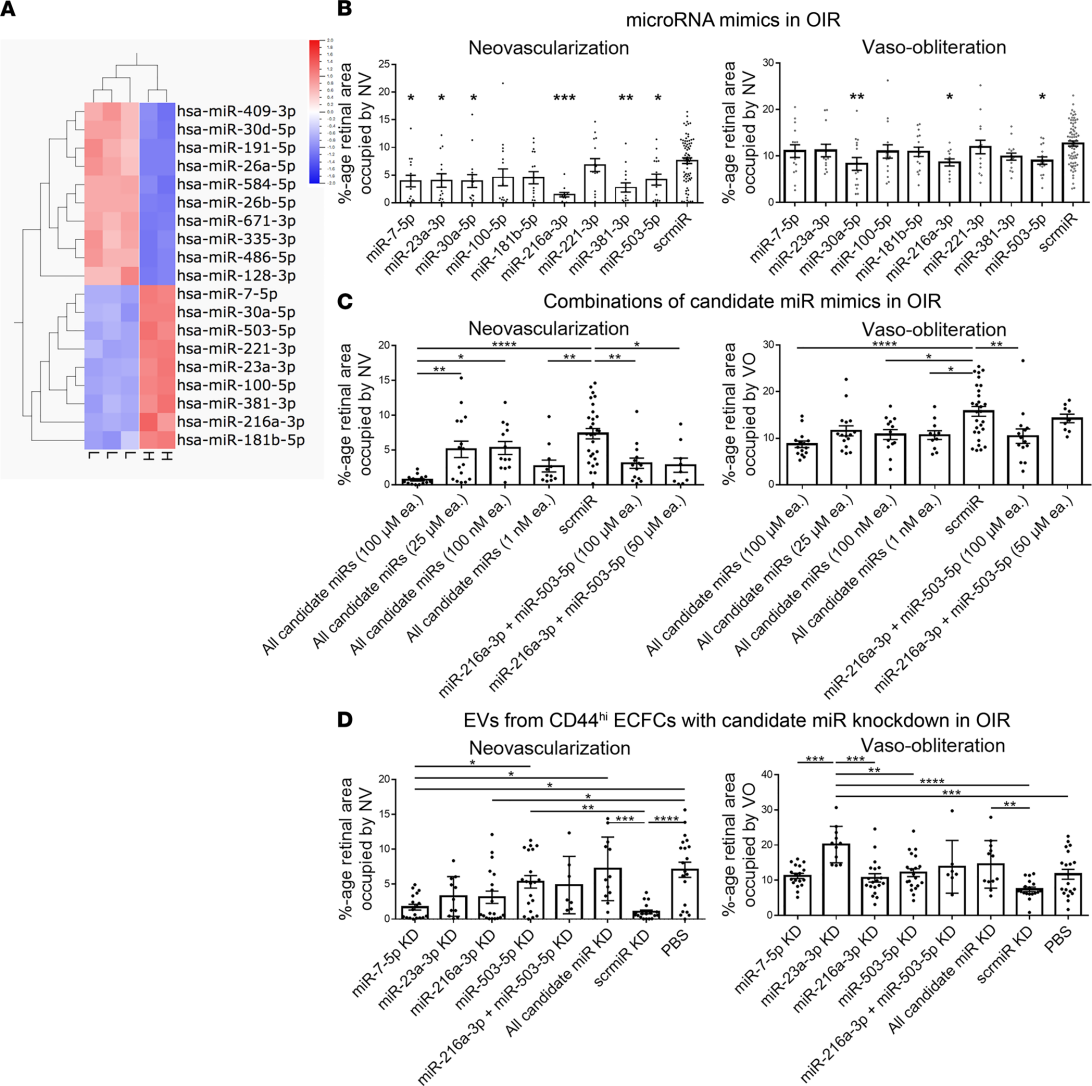
Differentially expressed miRs are neurovasculotrophic and contribute to the in vivo rescue effects of EVs^hi^. (**A**) Heatmap of differentially expressed (*q* < 0.05) miRs on small RNA sequencing of EVs^hi^ (H, *n* = 2) and EVs^lo^ (L, *n* = 3). (**B**) Injection of miR mimics upregulated in EVs^hi^ in **A** rescued NV (miR-7-5p, miR-26a-3p miR-30a-5p, miR-216a-3p, miR-381-3p, miR-503-5p) and VO (miR-30a-5p, miR-216a-3p, miR-503-5p) compared to scrmiR-injected controls. *n* = 12–16 retinas for miR mimics, *n* = 72 retinas for scrmiR. (**C**) Combinatorial injection of miR mimics rescued OIR mice dose-dependently. “Candidate miRs” miR-7-5p, miR-23a-3p, miR-216a-3p, and miR-503-5p were upregulated on small RNA sequencing in **A**, validated on RT-qPCR, and functional in rescuing OIR mice in **B**. Combination injection of the 2 most effective miR mimics (miR-216a-3p and miR-503-5p) in **B** and, separately, all candidate miR mimics rescued OIR mice. *n* = 9–14 retinas for miR-216a-3p and miR-503-5p, *n* = 11–16 retinas for all candidate miRs, *n* = 24 retinas for scrmiR. (**D**) EVs from CD44^hi^ ECFCs with KD expression of individual or combinatorial miRs no longer rescued OIR mice. Multiple lines of ECFCs were generated with KD expression of both miR-216a-3p and miR-503-5p, all candidate miRs together, and each candidate miR individually. EVs from miR-23a-3p KD CD44^hi^ ECFCs failed to rescue VO; EVs from miR-503-5p KD CD44^hi^ ECFCs failed to rescue NV; and EVs from CD44^hi^ ECFCs with KD expression of all candidate miRs failed to rescue both NV and VO in OIR mice. *n* = 11–20 retinas for individual miR KD, *n* = 7 retinas for miR-216a-3p and miR-503-5p KD, *n* = 12 for all candidate miR KD, *n* = 18 for scrmiR KD, *n* = 20 for PBS. One-way ANOVA with Tukey’s analysis. Error bars represent SEM. *P < 0.05, **P < 0.01, ***P < 0.001, ****P < 0.0001.

To distinguish which miRs may mediate rescue by EVs^hi^, comparative small RNA sequencing between EVs^hi^ and EVs^lo^ was employed to identify differentially expressed miRs in EVs^hi^. A heatmap of differentially expressed miRs (*q* < 0.05) is displayed in Figure 7A. A total of 9 miRs (miR-7-5p, miR-23a-3p, miR-30a-5p, miR-100-5p, miR-181b-5p, miR-221-3p, miR-216a-3p, miR-381-3p, miR-503-5p) were upregulated and 10 miRs were downregulated (miR-26a-5p, miR-26b-5p, miR-30d-5p, miR-128-3p, miR-191-5p, miR-335-5p, miR-486-5p, miR-409-3p, miR-584-5p, miR-671-3p) in EVs^hi^, compared to EVs^lo^. All upregulated miRs were validated on RT-qPCR, except miR-30a-5p and miR-381-3p (Supplemental Table 1). For functional validation, OIR mice were treated with mimics of differentially expressed miRs in EVs^hi^ to evaluate their innate neurovasculotrophic capacity. Compared with eyes injected with negative control scramble miR mimic (scrmiR), 6 miRs upregulated in EVs^hi^ (miR-7-5p, miR-23a-3p, miR-30a-5p, miR-216a-3p, miR-381-3p, miR-503-5p) rescued NV, and 3 of those miRs (miR-30a-5p, miR-216a-3p, miR-503-5p) also rescued VO (Figure 7B). Together, miR-7-5p, miR-23a-3p, miR-216a-3p, and miR-503-5p were defined as “candidate miRs” since they were differentially expressed on small RNA sequencing, validated on RT-qPCR, and trophic in OIR mice. To evaluate the effect of combinatorial miR treatment on OIR, all candidate miR mimics and, separately, the 2 most neurovasculotrophic miR mimics in OIR mice (miR-216a-3p and miR-503-5p) were injected into the model at various concentrations. Combinatorial injection of all candidate miR mimics as well as combinatorial miR-216a-3p and miR-503-5p injections rescued OIR mice in a dose-dependent manner (Figure 7C).

### KD of candidate miRs attenuates EVs^hi^ function in vivo.

To obviate the contribution of these 4 candidate miRs to the efficacy of EVs^hi^, EVs from CD44^hi^ ECFCs transfected with antisense oligonucleotides to reduce expression of these miRs were injected into OIR mice along with another negative control line of EVs from ECFCs transfected to KD expression of scrmiR. KD efficiency was 62.9% for miR-7-5p–KD ECFCs, 9.0% for miR-23a-3p–KD ECFCs, 25.3% for miR-216a-3p–KD ECFCs, and 48.3% for miR-503-5p–KD ECFCs by RT-qPCR. One cell line was generated with knocked-down expression of both miR-216a-3p and miR-503-5p at KD efficiencies of 11.7% and 19.1%, respectively. Another line was generated with KD expression of all candidate miRs with an efficiency of 33.7% for miR-7-5p, 35.9% for miR-23a-3p, 44.3% for miR-216a-3p, and 34.9% for miR-503-5p.

EVs were isolated from CD44^hi^ ECFCs with miR KD and injected into OIR mice. Relative to mice treated with EVs from CD44^hi^ ECFCs transfected with scrmiR, OIR mice treated with EVs from miR-7-5p–KD CD44^hi^ ECFCs and miR-216a-3p–KD CD44^hi^ ECFCs demonstrated slightly reduced rescue effects that were not statistically significant. Relative to EVs from scrmiR-transfected CD44^hi^ ECFCs, EVs from miR-23a-3p–KD CD44^hi^ ECFCs demonstrated significantly reduced rescue of VO; EVs from miR-503-3p–KD CD44^hi^ ECFCs no longer rescued both NV and VO; and EVs from CD44^hi^ ECFCs with KD of both miR-216a-3p and miR-503-5p trended toward reduced rescue, but these effects were not statistically significant. EVs from CD44^hi^ ECFCs with KD of all candidate miRs failed to rescue both NV and VO (Figure 7D).

## Discussion

An extension of the CNS, the neurosensory retina exhibits extreme metabolic demands that are met by an organized vascular architecture consisting of 3 vascular plexi and the choriocapillaris sandwiched between layers of neurons interspersed with various glial cell types. Endothelial and glial cells secrete factors critical in regulating homeostasis and maintaining a neurovascular stem cell niche (4). Trophic neurovascular crosstalk between vascular endothelial cells and other cells of the NVU lends merit to the concept of harnessing endothelial cells and their progenitors to rescue surrounding neurons under stress due to hypoxia or genetically encoded cell-specific mutations causing neurodegeneration. True endothelial progenitor cells, ECFCs stabilize atrophying retinal vasculature under hypoxic stress, protect retinal neurons from undergoing apoptosis, and, thus, serve as a promising cell source of therapy for ischemic/neurodegenerative CNS diseases (12, 16).

In this study, we hypothesized that EVs^hi^, as shuttles of neurovasculotrophic miRs, represent paracrine mediators capable of recapitulating the therapeutic effects of the bioactive CD44^hi^ ECFC population. We present multiple lines of evidence in support of this hypothesis. First, we demonstrated that EVs^hi^ provided potent therapeutic effects in neuroischemic/neurodegenerative mouse models by attenuating neovascularization, promoting vascular growth, and rescuing neural cell loss and function in OIR and RD10 mice. When exosome shedding was pharmacologically inhibited by GW4869, CD44^hi^ ECFCs no longer rescued the OIR model. Second, our data corroborated previous reports that EVs from ECFCs assume perivascular positions and colocalize within macrophages and microglia (20). EVs^hi^ also accumulated within neovascular tufts in the OIR model. Third, we demonstrated that neurovasculotrophic miRs differentially expressed within EVs^hi^ were required for their effects. KD of miR expression in CD44^hi^ ECFCs attenuated the rescue effects of cells and their EVs. Small RNA sequencing identified dysregulated miRs between EVs^hi^ and EVs^lo^. The bioactivity of upregulated miRs in EVs^hi^ was tested by injecting miR mimics into OIR mice. Four intravesicular miRs (miR-7-5p, miR-23a-3p, miR-216a-3p, miR-503-5p) were defined as candidate miRs since they were upregulated in EVs^hi^ on small RNA sequencing, were validated on RT-qPCR, and were neurovasculotrophic in the OIR model. EVs from CD44^hi^ ECFCs with miR-23a-3p or miR-503-5p KD failed to rescue the OIR phenotype. Similarly, EVs from ECFCs with combinatorial KD of all candidate miR mimics failed to rescue OIR mice. Together, these data suggest that EVs^hi^ represent a promising therapeutic agent for the treatment of ischemic and neurodegenerative retinopathies through a mechanism partially dependent on intravesicular miR composition.

ECFC-derived EVs have demonstrated therapeutic activity in numerous angiogenesis-dependent animal disease models, including diabetic wound healing (33), re-endothelialization of vascular injury (34), traumatic brain injury (35), and osteogenesis (36). A mechanistic role for intravesicular miR has been demonstrated in ECFC EV–mediated repair of ischemic animal models, including acute kidney injury (37–41), hind limb ischemia (42), and sepsis (43).

The current study adds to this literature by demonstrating that EVs^hi^ are the bioactive subset of vesicles that rescue models of CNS ischemia and neurodegeneration through a mechanism dependent on intravesicular miR-23a-3p, miR-503-5p, and a combination of neurovasculotrophic miRs. In models of ischemic and/or traumatic brain injury, miR-23a-3p decreases oxidative stress, neuron apoptosis, and neural inflammation to improve vascular and neuronal outcomes (44, 45). The role of miR-503 as an antiangiogenic agent is consistent in vitro and in vivo across literature (46). In cancer models, miR-503 inhibits tumor angiogenesis by downregulating vascular growth factors, particularly VEGF-A, and is downregulated by hypoxia-induced expression of HIF1α (47–49). VEGF-A is overexpressed in many ischemic/neurodegenerative CNS diseases, and modulation of VEGF-A via therapeutic agents that restore miR-503-5p, such as EVs^hi^, is a promising treatment strategy (50). Our prior study demonstrated that CD44^hi^ ECFCs achieve their therapeutic effects in OIR mice through a mechanism partially mediated by IGFBP2 and IGFBP3 (12). Together, these data support the hypothesis that the diverse bioactive cargo secreted by CD44^hi^ ECFCs and within their shed EVs may initiate multiple mechanisms within multiple target cell types simultaneously to provide neurovasculotrophic support to the ischemic/neurodegenerative retina.

Several practical limitations must be addressed to facilitate translation of EV-based therapeutics to the clinics. An EV isolation protocol capable of isolating “pure” samples with a low soluble protein/EV ratio must be scalable to meet clinical demands. Comparative reports have demonstrated that SEC-based EV isolation protocols produce EV samples that are superior in purity, integrity, and functionality relative to the majority of other methods (51–56). The UF-SEC-UF protocol developed in this study significantly improved the yield and bioactivity of EVs while effectively isolating pure EV samples suitable for use in downstream applications (21, 57, 58); the final concentration of the EV sample can be controlled by the last UF step. The observation that EVs^hi^ from any healthy UCB donor rescued OIR mice supports the concept that EVs^hi^ may be efficiently derived from any healthy donor or banks of pooled UCB. EVs^hi^ also demonstrated a favorable storage profile. Although most EVs were prepared fresh before injection, samples frozen at –80°C for as long as 1 year retained therapeutic function in OIR mice. Early EVs^hi^ treatment at the onset of the ischemic drive rescued OIR mice whereas injection of EVs^hi^ during neovascularization was ineffective. Ultimately, treatment timing must be determined by human clinical trials. EVs^hi^ demonstrated a favorable safety profile; treatment promoted physiologic vascular growth during the vaso-obliterative phase of OIR but did not affect developing vasculature of newborn pups.

In conclusion, the bioactive subset of CD44^hi^ ECFCs shed therapeutic EVs^hi^ loaded with miRs that, at least in part, mediated neurovasculotrophic effects in models of neuroischemia/neurodegeneration in brain tissue. These results suggest that EVs^hi^ loaded with trophic miRs are a promising therapeutic agent for the treatment of ischemic and neurodegenerative retinal diseases as well as other diseases of the CNS.

## Methods

### Animals.

OIR was induced in C57BL/6J mice (The Jackson Laboratory, JAX) as previously described (12). Briefly, pups and their mothers were transferred from room air to a hyperoxic (75% O_2_) chamber (Bio-Spherix) on P7 for 5 days, during which the hyperoxic environment leads to VO of central retinal blood vessels. Pups were returned to room air and transferred to a surrogate mother on P12, and the resulting ischemia stimulated an NV phase characterized by the formation of preretinal neovascular tufts, which peaked at P17. Pde6b^rd10/rd10^ RD10 mice (B6.CXB1-Pde6b^rd10^/J) were purchased from JAX. C57BL/6J pups were used for vascular developmental models.

### Cell preparation and culture.

Human UCB was obtained following full-term gestation from healthy, nondiabetic donors and was processed within 2 hours of delivery in all cultures. ECFCs were derived from 4 UCB donors and cultured as previously described (12). In brief, UCB (40–60 mL) was diluted 1:2 in PBS with 20 μg/mL heparin (STEMCELL Technologies, 07980), and mononuclear cells were isolated using Lymphoprep (STEMCELL Technologies, 07851). After 3 PBS washes, mononuclear cells were resuspended in EC-Cult XFM (STEMCELL Technologies, 0800) and seeded at a density of 50 × 10^6^ cells/well onto 6-well plates coated with ACF cell attachment substrate (STEMCELL Technologies, 07130). After daily medium changes for the first 7 days, medium was changed every other day. Passage 7 ECFCs were used for experiments. HUVECs (Lonza, C2519A) were cultured using M200 medium (Thermo Fisher Scientific, M200500), and passage 2 cells and their EVs were used in experiments in OIR mice.

### ECFC immunophenotyping and sorting.

The immunophenotype of ECFCs was determined by flow cytometry on 3 biological replicates of ECFCs using fluorescence-conjugated antibodies. ECFCs were sorted into CD44^hi/lo^ populations as previously described (12). Briefly, cells were detached (Animal Component-Free Cell Dissociation Kit, STEMCELL Technologies, 05426) and PBS washed. ECFCs were incubated for 20 minutes on ice with 20 μL of APC-conjugated primary murine monoclonal antibodies against human CD44 antibody (clone G44-57, catalog 559942, BD Pharmingen) in 0.4 mL stain buffer (PBS [Dulbecco’s, no Ca^2+^, no Mg^2+^, Thermo Fisher Scientific, 14190250], 5% FBS, with 0.5 mM EDTA), washed 3 times, and analyzed by FACSAria flow cytometer (BD) with FlowJo (TreeStar) software. Duplicate and dead cells were excluded from the sort using forward and side scatter to analyze their 2-dimensional profile. Fluorescence voltages were set using negative controls, and the same strategy for setting parameters and gating were applied to all samples. The same staining protocol was applied for all FACS analyses of ECFCs using antibodies and concentrations listed in Supplemental Table 2, and the same gating strategy was used for all cell sorting experiments.

### EV isolation.

EVs were isolated from ECFC CM as previously described with modifications (21). Serum-free XFM (10 mL/T75 flask) was conditioned for 48 hours by sorted passage 7 CD44^hi/lo^ ECFCs seeded at a density of 1.2 × 10^6^ cells/T75 flask (Thermo Fisher Scientific, 07-202-000). Pooled CM was removed of cell debris via centrifugation at 300*g* for 5 minutes (Beckman Coulter Alegra 6KR, ARIES Smart Balance Rotor), and supernatant was 0.22 μm vacuum filtered (Corning, 430320). CM was loaded into an Amicon Ultra-15 Centrifugal Filter Unit with an Ultracel-10 membrane (MWCO = 10 kDa; Merck Millipore, UFC901024) for UF to concentrate CM to 1 mL via centrifugation at 4000*g* for approximately 45 minutes (Beckman GS-6R, GH-3.8 swing bucket rotor). The concentrated sample was then subjected to SEC as previously described with some modifications (58). For the SEC column, the tip of a 10 mL plastic syringe (BD, 309604) was packed with nylon stocking (20 denier, H&M) and loaded with 10 mL sepharose CL-2B (GE Healthcare, 17014001) that was washed beforehand with elution buffer (PBS with 0.32% trisodium citrate, pH 7.4, 0.22 μm vacuum filtered) 3 times. The column was run dry before UF-concentrated CM was loaded. Once the CM entered the stationary phase, 20 mL elution buffer was slowly added, and eluate was collected in approximately 40 sequential EFs of 0.5 mL each. Soluble protein of each EF was measured using a Pierce BCA protein assay (Thermo Fisher Scientific, 23225) according to the manufacturer’s instructions. Protein began eluting between fractions 8 and 12. All EFs prior to protein detection were pooled and concentrated to 250–500 μL using an Amicon Ultra-4 Centrifugal Filter Unit with an Ultracel-10 membrane (MWCO = 10 kDa; Merck Millipore UFC801024) by centrifugation at 4000*g* for approximately 45 minutes to produce an EV-rich, soluble protein–poor UF-SEC-UF sample. This sample was measured with nanoparticle tracking analysis (NTA) and aliquoted for experiments either fresh or after storage at –80°C. EVs harvested using differential UC were isolated as previously described using a Beckman Coulter Optima L-80 XP ultracentrifuge (57). EV yield was calculated by multiplying the particle concentration on NTA by the final sample volume, divided by the number of T75 flasks from which CM was pooled. Experiments in OIR mice were performed with EVs from at least 4 biologically independent ECFC colonies derived from distinct UCB donors and from 2 distinct UCB donors in RD10 mice. EVs from HUVECs were obtained from 2 independent UF-SEC-UF isolation protocols from a single population of expanded cells.

### ECFC transfection.

CD44 expression was knocked down in ECFCs using green fluorescent protein (GFP) lentiviral shRNA clones to CD44 (CMV-Neo, GenTarget Inc., clones TRCN0000308110 and TRCN0000296190) to generate ECFCs-shCD44. ECFCs-shDICER1 were also generated using lentiviral shRNA clones (Santa Cruz Biotechnology, sc-40489-V). Cell transfections were performed in accordance with the manufacturer’s protocol with antibiotic selection with puromycin (1 μg/mL media for ECFCs-shCD44 and 5 μg/mL media for ECFCs-shDICER1, Santa Cruz Biotechnology, sc-108071). Two separate lines of ECFCs-scrRNA were transfected with scrRNA to serve as negative control cells in ECFCs-shCD44 experiments (GenTarget Inc.) or ECFCs-shDICER1 experiments (Santa Cruz Biotechnology, sc-108080). KD of miR-7-5p, miR-23a-3p, miR-216a-3p, and miR-503-5p in ECFCs was achieved using miRCURY LNA miRNA Power Inhibitors with fluorescein amidite labeling and the corresponding scrmiR Negative Control A (YI04100814-DDB, YI04103406-DDC, YI04104404-DDB, YI04100899-DDB, and YI00199006-DDB, respectively, QIAGEN, 1 μM). Transfection efficiency was measured as the average percentage DAPI^+^GFP^+^ cells in 5 sample confocal images of ECFCs-shCD44 or ECFCs-scrRNA fixed in 4% paraformaldehyde for 20 minutes. Transduction efficiencies were measured on RT-qPCR.

### EV characterization.

For magnetic bead–assisted flow cytometry, Exo-Flow Capture Kits (Systems Biosciences) were used in accordance with the manufacturer’s protocol (Supplemental Table 2) to confirm the presence of vesicles in all EV samples. Briefly, magnetic, 9.1 μm, streptavidin-coated beads were washed twice and incubated with 10 μL biotinylated CD9, CD63, CD81, or CD31 capture antibody for 2 hours at 4°C with gentle agitation every 30 minutes. Following 3 washes, beads were incubated with EV samples in a rotating rack at 4°C overnight for exosome capture. Beads were then washed twice before incubation with 10 μL of proprietary Exo-FITC antibodies for 2 hours on ice. After 3 additional washes, beads were suspended in wash buffer and analyzed by flow cytometry. A DynaMag-2 (Thermo Fisher Scientific, 12321D) stand was used to magnetically precipitate beads during wash steps. NTA was used on all EV samples to measure particle concentration. EV samples were diluted in Dulbecco’s PBS (Dulbecco’s no Ca^2+^/Mg^2+^) in a cuvette analyzed on the ViewSizer 3000 (HORIBA Scientific). Upon illumination with blue (450 nm at 210 mW), green (520 nm at 12 mW), and red (635 nm at 8 mW) lasers, 50 videos (30 seconds each, exposure: 15 ms) of Brownian motion of nanoparticles were recorded and analyzed by ViewSizer software to determine particle size distribution and concentration. For TEM, negative stains of EV samples were imaged by a CM100 FEI electron microscope (Philips) at an 80 kV accelerating voltage. First, a Formvar-carbon-coated grid (MilliporeSigma) was loaded with 8 μL of sample for 2 minutes to allow for adsorption. Excess sample was removed from the grid using a clean filter paper. The coated side of each grid was placed facedown in a droplet of phosphotungstic acid (MilliporeSigma) for 2 minutes, and then samples were imaged. For CM-DiI staining, EVs^hi^ were incubated with CM-DiI (Thermo Fisher Scientific, C7001, 1 μM) for 5 minutes at 37°C, for 20 minutes on ice, and then were washed twice with PBS spins at 100,000*g* immediately prior to injection into OIR mice.

### Intravitreal injections.

Mice were injected intravitreally using a 33-gauge needle (Hamilton). All injections were 0.5 μL in volume. Intact CD44^hi^ or CD44^lo^ ECFCs and HUVEC controls were injected along with PBS vehicle controls. Exosome biogenesis and release from CD44^hi^ ECFCs was pharmacologically inhibited by 1-hour incubation with the neutral sphingomyelinase inhibitor GW4869 (20 μM) dissolved in DMSO (+GW4869) whereas control CD44^hi^ ECFCs were incubated with an equal volume of DMSO (–GW4869). All cells were injected at a concentration of 1 × 10^5^ cells/μL in PBS vehicle. Following measurement of EV concentration using NTA, 1 × 10^5^ EV particles/0.5 μL/eye were injected into OIR and RD10 mice. EVs from HUVECs served as an EV control. PBS served as a vehicle control. To ensure media components were not responsible for observed effects, nonconditioned media for ECFCs (XFM) and HUVECs (M200) was subject to the same UF-SEC-UF EV isolation protocol and injected into OIR mice. EVs^hi^ sample was vesicle-depleted by 18 hours of UC at 120,000*g* and injected into OIR mice. ECFC-derived EVs isolated in a total of 9 UF-SEC-UF preps from media conditioned by each of 4 distinct biological UCB donors were tested in at least 3 litters of OIR mice. In RD10 mice, ECFC-derived EVs from 2 individual UCB donors promoted functional rescue of the neural retina on electroretinography. MicroRNA mimics of miR-7-5p, miR-23a-3p, miR-216a-3p, and miR-503-5p (Thermo Fisher Scientific, mirVana mimics, MC11755, MC10644, MC24316, MC10378, respectively) as well as scrmiR (Thermo Fisher Scientific, mirVana mimic, Negative Control 1, 4464085) were intravitreally injected into OIR mice.

### Immunofluorescence.

To prepare retinal flat mounts, enucleated eyes were fixed in 4% paraformaldehyde for 1 hour at 4°C. The anterior capsule was removed to allow for dissection of the nuclei and cortex of the lens. The retina was separated from choroid and sclera, cleaned of remaining vitreous with fine brushes, and cut into 4 leaflets. Dissected retinas were incubated overnight in PBS with Ca^2+^Mg^2+^ with 10 μg of fluorescently labeled GS-IB4 (Thermo Fisher Scientific, I21412). For retinal cryosections, eyes fixed for 4 hours were punctured at the limbus with small forceps and incubated in 30% sucrose overnight prior to freezing in OCT media–filled (Thermo Fisher Scientific) molds for sectioning. For antibody staining, retinas were rehydrated with PBS prior to overnight incubation with gentle rocking at 4°C in block buffer (PBS with 10% fetal calf serum, 10% serum matching the host species of the primary antibody, and 0.3% [v/v] Triton X-100 [MilliporeSigma, T8787]). Primary antibodies used in this study are listed in Supplemental Table 2. Following 5 washes in PBS for 10 minutes each, retinas were incubated with corresponding fluorescently labeled Alexa secondary antibodies (Thermo Fisher Scientific, A-11008) in block buffer with 0.1% (v/v) Triton X-100 overnight in 4°C with gentle rocking. Nuclei were stained with Hoechst 33342 (Thermo Fisher Scientific, 62249) or DAPI (Thermo Fisher Scientific, 62248). In each staining protocol, retinas were washed 4 times in PBS for 10 minutes before mounting with SlowFade Gold Antifade Mountant medium (Thermo Fisher Scientific, S36937). TUNEL staining was performed using an In-Situ Cell Death Detection Kit (Roche Diagnostics, 11684795910) according to the manufacturer’s instructions.

### Confocal microscopy and quantification.

Retinas were imaged using a Zeiss 710 confocal laser-scanning microscope with ZEN 2010 software (Zeiss). The percentage retina covered by NV and VO in OIR mice was quantified with our published deep learning algorithm (59). In cases where quantification using this algorithm disagreed with expert inspection of the images, reported measurements were quantified using manual quantification methods as previously described (60). Briefly, the lasso tool in Photoshop CS6 (Adobe) was used to outline and record total retinal area, and the VO was measured by tracing the central avascular retina; the magnetic lasso tool was used to highlight NV. VO was quantified manually for experiments injecting OIR mice on P7 for quantification on P10, P12, and P14. Vascular coverage in developmental models was quantified as the traced area of vascular coverage divided by the total retinal area traced using the lasso tool in Photoshop. For quantification of retinal thicknesses in OIR eyes, the INL, IPL, and ONL were measured in stitched 20× original magnification images of retinal cross sections, and the averaged thickness at preselected distances from the optic nerve was reported. For quantification of vascular plexi in RD10 mice, 8 *Z*-stack images (4 central and 4 peripheral) at 20× original magnification (326 × 326 μm fields of view) were taken of each retina of P21, P25, P32, P40, and P60 mice. The images in focus on the deep and intermediate vascular plexi were selected for quantification at each location from the *Z*-stack. For each eye, the total vessel area and length quantified using AngioTool (61) (NIH) were reported as the averages from these 8 images per retina. The number of branching points was manually quantified in ImageJ (NIH). To quantify retinal thickness and density of TUNEL-positive cells in the ONL, a series of 20× original magnification images of retinal cross sections were acquired. To measure the density of apoptotic cells, the number of apoptotic TUNEL^+^DAPI^+^ cells was divided by the ONL area as calculated in ImageJ as previously described (12). ONL thickness was measured at selected distances from the optic nerve using ImageJ as previously described (62). Per eye, quantification of the percentage retinal area covered by cone photoreceptors was performed on 4 representative 20× original magnification images measured 500 μm from the optic nerve of red/green opsin-stained retinal flat mounts. The percentage area of opsin-positive pixels was obtained via threshold selection of grayscale images in ImageJ, and results were normalized to those from normoxic retinas.

### Ganzfeld ERG.

ERG was performed as previously described (12). Mice were dark-adapted overnight. Anesthesia was administered via intraperitoneal injection of 20 mg/mL ketamine and 2 mg/mL xylazine at a dose of 5 μL/g body weight. Following pupil dilation with 2.5% phenylephrine and 1% tropicamide, full-field ERGs using silver needle electrodes as reference (forehead) and ground (tail) were measured from the corneal surface using active contact lens electrodes (Mayo). Conditions were controlled via a Ganzfeld dome using Espion E2 computer software (Diagnosys). Dark-adapted (scotopic) recordings were made of rod responses to a series of white light flashes of increasing intensities (25 and 50 cd × s/m^2^ reported). Light-adapted (photopic) conditions were induced by a 30 cd/m^2^ background luminescence for 5 minutes, and measurements were made on cone responses to a single flash (intensity 25 cd × s/m^2^) as well as to 1 Hz flicker stimuli. For all ERG measurements, responses were filtered at 0.3–500 Hz and averaged signals were reported. To circumvent the observation that ERG measurements can vary significantly between litters of untreated RD10 mice (data not shown) and the possible bias imposed by eye dominance, treatment groups were randomized within each litter, and both eyes were injected with the same treatment per mouse. ERG experiments investigating the effect of EVs from sorted CD44^hi/lo^ ECFCs were conducted separately from ERG experiments injecting EVs from ECFCs-shCD44 or EVs versus ECFCs-scrRNA.

### Small RNA sequencing.

For exosomal RNA extraction, RNA was extracted from EVs^hi^ and EVs^lo^ using the Plasma/Serum (PS) Circulating and Exosomal RNA Purification Mini Kit (Norgen BioTek, 51000) as previously described (63). Samples were incubated for 10 minutes at 60°C with 100 μL warmed PS Solution A and 900 μL warmed PS Solution B (containing 2-Mercaptoethanol). After adding 1.5 mL of 100% ethanol, samples were centrifuged for 30 seconds at 100*g*. The pellet was resuspended in 750 μL PS Solution C and incubated again for 10 minutes at 60°C. After adding 750 μL of 100% ethanol, this solution was loaded onto the filter column and centrifuged for 1 minute at 16,000*g*. Following 3 wash spins for 1 minute at 16,000*g* using 400 μL Wash Solution, the column was centrifuged again to dry the membrane. The column was loaded with 30 μL water, and a slow spin for 2 minutes at 300*g* followed by a fast spin for 3 minutes at 16,000*g* eluted the RNA. For library preparation and small RNA sequencing, libraries for small RNA sequencing were constructed using the NEBNext Small RNA Library Prep Set for Illumina as previously described (63). Reactions were conducted at one-fifth the suggested volume and adaptors at one-sixth the provided concentration with 18 PCR cycles. Libraries were prepared from 1.2 μL of RNA for each sample. A Zymo DNA Clean & Concentrator Kit (Zymo Research, D4013) was used to clean library product. Libraries were pooled based on PicoGreen measurements of concentration and the proportion of the desired PCR product and adaptor dimers were observed using a Fragment Analyzer high-sensitivity DNA array (Advanced Analytical). Pooled libraries were size selected to remove adaptor dimers using the Pippin Prep HT instrument (Sage Science) with the lower limit of size selection set to 125 and the upper limit set to 150. Size selected libraries were sequenced on a MiSeq instrument (Illumina) for initial analyses and quality control before samples were sequenced on an Illumina HiSeq 4000 as 50-cycle single-end reads. For analysis of small RNA sequencing data, all-pass-filtered miRs with more than 10 reads in any 1 sample in each group were included for analyses. Qlucore Omics Explorer was used for principal component analysis, hierarchical clustering, and data visualization. Differential expression analysis was also performed by the Qlucore Omics Explorer using the “Two Group” comparison tool. To identify miRs possibly responsible for the therapeutic effects of EVs^hi^, we elected to investigate differentially expressed miRs by thresholding with *q* < 0.05, which helps circumvent the false positive rate inherent in thresholding large numbers of parameters with *P* < 0.05 and Bonferroni’s correction.

### RT-qPCR.

To measure ECFC transduction efficiency, total RNA was isolated from cells using the RNeasy Micro Kit (QIAGEN, 74004) and reverse-transcribed using the High-Capacity RNA-to-cDNA Kit (Thermo Fisher Scientific, 4388950). The CFX96 Touch Real-Time PCR Detection System (Bio-Rad) was used to perform RT-qPCR using TaqMan Gene Expression Assays or TaqMan MicroRNA Assays for measurements of lentiviral transduction efficiency. Validation of small RNA-sequencing data was achieved using TaqMan Advanced MicroRNA Assays targeting differentially expressed miRs according to the manufacturer’s instructions. All materials used for RT-qPCR are listed in Supplemental Table 3. The housekeeping genes used for normalization of RT-qPCR data were beta-actin for DICER1-KD cells, GAPDH for CD44-KD cells, and snU6 for all miR-KD cells.

Analyses were performed as previously described with modifications (64). Normalization of extracellular miR data sets has proved to be challenging. Standard approaches to normalization like using spike-in synthetic oligonucleotides, housekeeping small RNAs, or bioinformatic techniques often applied in cellular long RNA-Seq data sets have not been successful. Studies of miRs within cells and tissues even advocate for use of sample set–specific normalizers (65), and the challenge of normalization in exosomal RNA data sets is commonly accepted (66). For pairs of endogenous miRs, the expression of each miR can serve as an endogenous control for the others, resulting in more reproducible features than the measured abundance of each individual miR (64). This paired normalization approach was first described in Price et al. and results in the formation of ratios of individual miR abundance (67). For our RT-qPCR validation of sequencing data, this technique was implemented to form ratios of each miR upregulated in EVs^hi^ to each miR downregulated in EVs^hi^ on small RNA sequencing to assess whether these expression trends can be validated.

### Statistics.

Prism (version 6, GraphPad Software) software was used for all statistical analysis. For experiments containing 2 groups, an unpaired, 2-tailed Student’s *t* test was used. One-way ANOVA with ad hoc Tukey’s analysis was used for multiple comparisons. For nonparametric data, a Kruskal-Wallis test with Dunn’s multiple-comparison test was performed. Statistical significance was determined as *P < 0.05, **P < 0.01, ***P < 0.001, ****P < 0.0001 in all figures.

### Study approval.

Experimental procedures using animals were approved by Scripps Research Animal Care and Use Committee. Experiments were performed in accordance with the NIH *Guide for the Care and Use of Laboratory Animals* (National Academies Press, 2011). In accordance with the Declaration of Helsinki, adult donors of UCB provided informed consent prior to sample collection. Protocols were approved by the Institutional Review Board at Scripps Research and Scripps Memorial Hospital, La Jolla, California, USA.

## Author contributions

KVM designed experiments, performed experiments, and wrote and edited the manuscript. EA, AUO, GW, and SS helped design and perform experiments. YI assisted in maintaining animal colonies. MF supervised the work and reviewed and edited the manuscript.

## Supplementary Material

Supplemental data

## Version 1 05/31/2022

In-Press Preview

## Version 2 06/22/2022

Electronic publication

## Version 3 06/29/2022

Guoqin Wei's and Yoichiro Ideguchi's names were corrected
