# Supplementary material for: Bioactive extracellular vesicles from a subset of endothelial progenitor cells rescue retinal ischemia and neurodegeneration
Source: JCI Insight. 2022 Jun 22;7(12):e155928. doi: 10.1172/jci.insight.155928 (PMC9309054; doi:10.1172/jci.insight.155928)
Supplement: Supplemental data [file jciinsight-7-155928-s275.pdf]

Supplemental Data

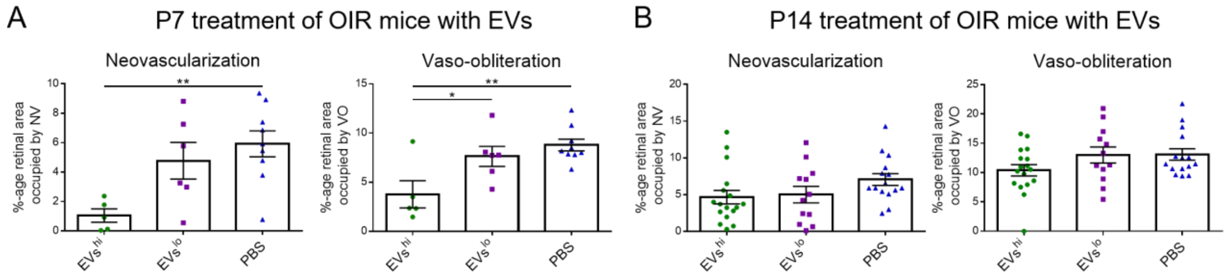

Supplemental Figure 1: **Pretreatment of OIR mice with EVs^{hi} rescued the model while treatment during the neovascular phase failed.**

(**A-B**) Quantification of NV and VO in mice treated with EVs^{hi}, EVs^{lo}, or PBS (on P7) prior to incubation in hyperoxia in **A** and (at P14) two days into the neovascular phase of the OIR model in **B**. 1-way ANOVA with Tukey analysis; P7 injections: n=5 retinas for EVs^{hi} n=6 retinas for EVs^{lo}, n=9 retinas for PBS; P14 injections: n=17 retinas for EVs^{hi} n=12 retinas for EVs^{lo}, n=15 retinas for PBS. Error bars represent SEM.

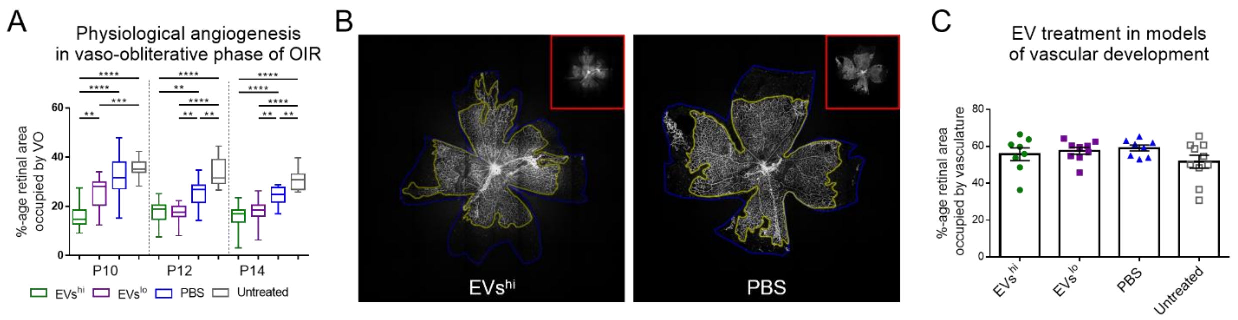

Supplemental Figure 2: **EVs^{hi} promoted vascular growth during the vaso-obliterative phase of OIR and had no effect in models of vascular development.** (**A**) Quantification of VO in retinal flat-mounts of OIR mice at P10, P12, and P14 during the vaso-obliterative phase following P7 injection demonstrated decreased VO in mice treated with EVs^{hi} in comparison to EVs^{lo}, PBS, or untreated controls. Data is represented as a Whisker plot where the top and bottom of the box represent mean of the upper and lower quartiles, the horizontal line within the box represents the mean, and the bars outside the box represent the min and max data point. 1-way ANOVA with Tukey analysis; n=11-13 retinas for EVs^{hi}, n=14-15 retinas for EVs^{lo}, n=13-16 retinas for PBS, n=16-21 retinas for untreated mice. (**B-C**) EV treatment does not affect vascular development. (**B**) Representative images of GS-IB4 lectin-stained retinal flat-mounts at P5 following P2 injection of wild type pups with EVs^{hi}, EVs^{lo}, PBS, or untreated controls (**C**) Quantification of the percentage of retinal area covered by vasculature demonstrated no effect of EV treatment on vascular development. 1-way ANOVA; n=8 retinas for EVs^{hi}, n=9 retinas for EVs^{lo}, n=8 retinas for PBS, n=10 retinas for untreated mice. Error bars represent SEM.

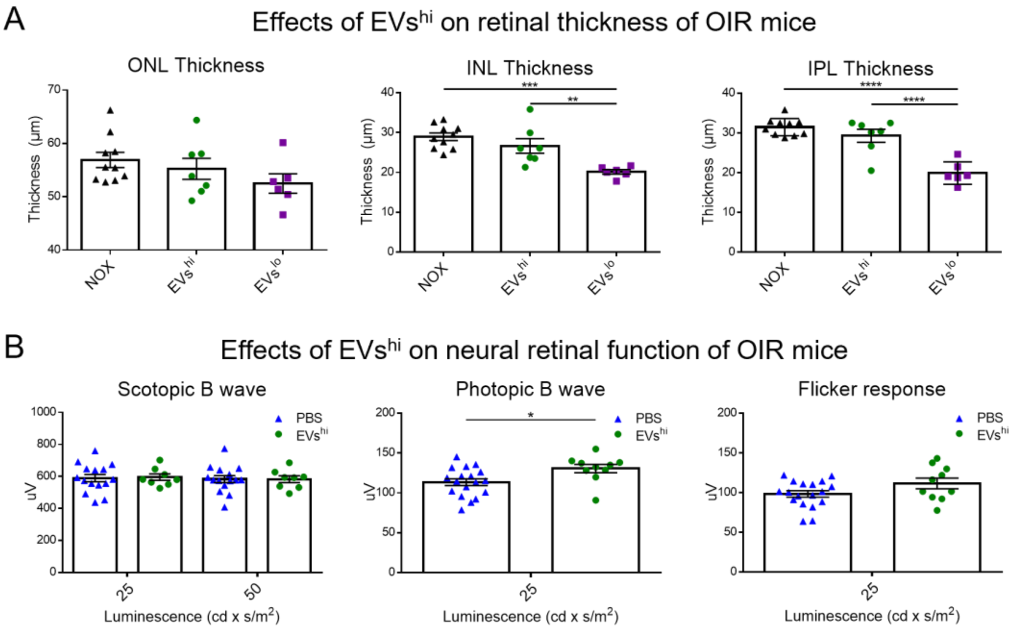

Supplemental Figure 3: **EVs^{hi} were neuroprotective in the OIR model.**

(**A**) Quantification of retinal thickness of the outer nuclear layer, inner nuclear layer, and inner plexiform layer at P30 following P12 treatment with EVs^{hi} versus EVs^{lo} as compared to normoxic (NOX) mice. Thicknesses were measured on retinal cross-sections. EVs^{hi} restored the INL and IPL (significantly greater than EVs^{lo}) to a thickness comparable to that of normoxic mice. 1-way ANOVA with Tukey analysis; n=7 retinas for EVs^{hi}, n=6 retinas for EVs^{lo}, and n=10 retinas for normoxic mice. (**B**) ERG recordings of neural retinal function measured on P30 following P12 treatment of OIR mice with EVs^{hi} demonstrated augmented photopic B wave compared to PBS vehicle-treated controls. Two-tailed Student's *t* test; n=8-10 retinas for EVs^{hi}, n=15-18 for PBS. Error bars represent SEM.

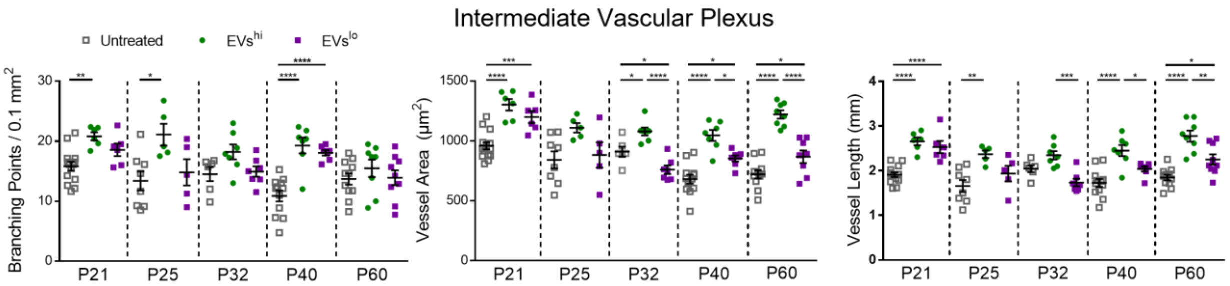

Supplemental Figure 4: **EVs^{hi} delayed atrophy of the intermediate plexus in inherited retinal degeneration mice.**

Quantification of the branching points (left), total vessel area (middle), and total vessel length (right) in the deep vascular plexus at P21, P25, P32, P40, and P60 demonstrated EVs^{hi} delay atrophy of the intermediate plexus. 1-way ANOVA with Tukey analysis; n=5-9 eyes in EV groups, n=8-14 eyes in untreated groups. Error bars represent SEM. *P<0.05, **P<0.01, ***P<0.001, ****P<0.0001

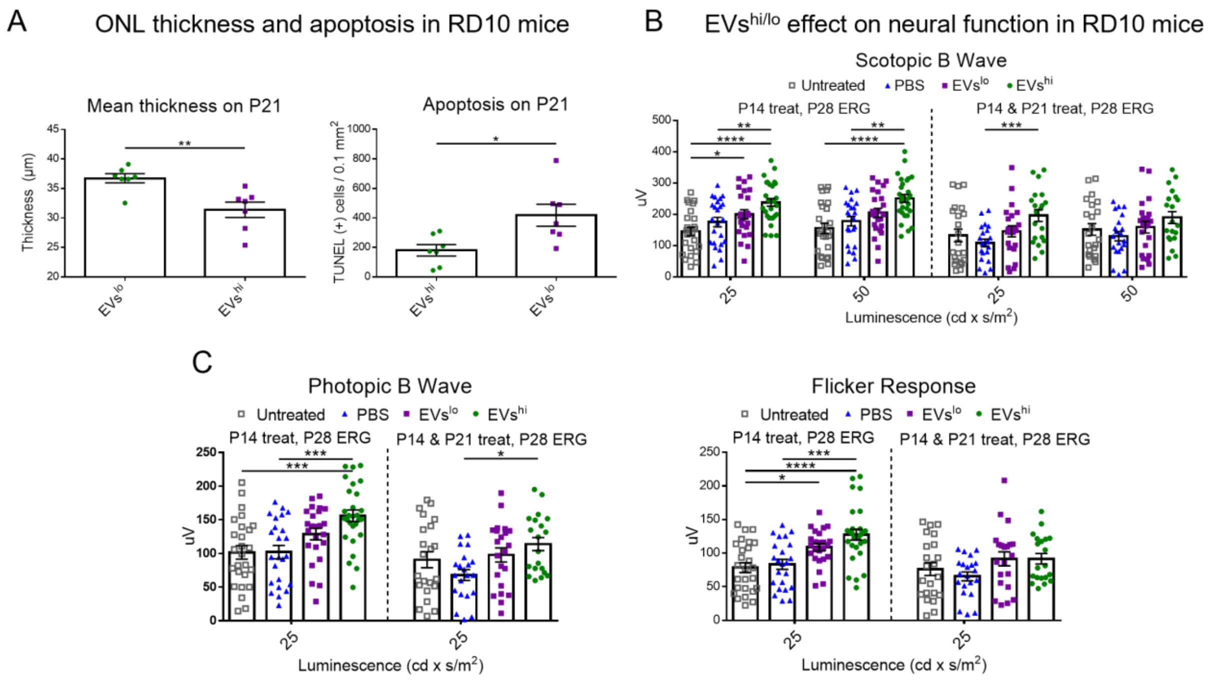

Supplemental Figure 5: **Repeated EVs^{hi} treatment may not augment neurotrophic support to inherited retinal degeneration mice.**

(**A**) Quantification of outer nuclear layer (ONL) thickness (left) and TUNEL staining (right) on immunohistochemistry of cross-sections from RD10 retinas treated P14 with EVs^{hi} or EVs^{lo} and harvested on P21. Two-tailed Student's *t* test; n=7 retinas for both groups. (**B-C**) Repeated injections are deleterious to retinal function of RD10 mice. ERG recordings of mice treated once (on P14, left of dotted line) versus twice (on P14 and P21, right of dotted line) demonstrated that repeated injections decreased retinal function in dark-adapted (scotopic) responses in **B** and light-adapted (photopic and flicker) responses in **C** across all groups. 1-way ANOVA with Tukey analysis; for experiments treating mice once at P14: n=28 retinas for EVs^{hi}, n=24 retinas for EVs^{lo}, n=23-24 retinas for PBS, n=25-26 retinas for untreated; for experiments treating mice twice at P14 and at P21: n=20 retinas for EVs^{hi}, n=21-22 retinas for EVs^{lo}, n=21 retinas for PBS, n=22 retinas for untreated. Error bars represent SEM.

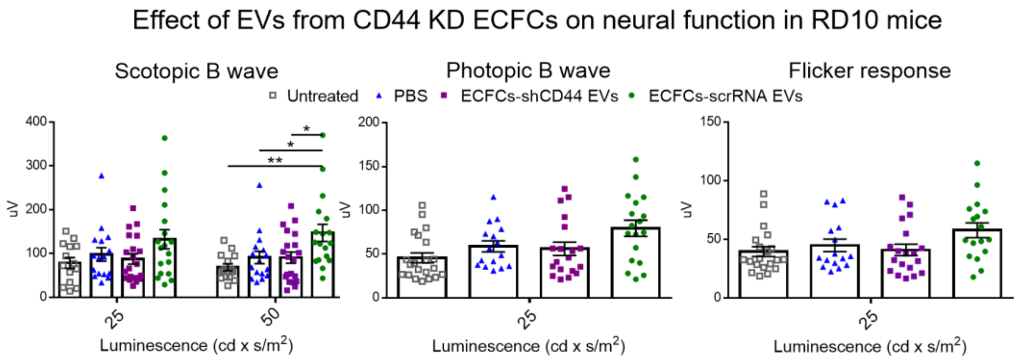

Supplemental Figure 6: **EVs from ECFCs with CD44 knockdown did not preserve neural retinal function in inherited retinal degeneration mice.**

EVs isolated from ECFCs transfected with shRNA to CD44 (ECFCs-shCD44 EVs) and from control ECFCs transfected with scramble RNA (ECFCs-scrRNA EVs) were injected into RD10 mice on P14 along with PBS and untreated controls for ERG measurements on P28. ECFCs-shCD44 EVs failed to rescue rod-driven dark-adapted responses (scotopic B wave), which were significantly improved by treatment with ECFCs-scrRNA EVs. 1-way ANOVA with Tukey analysis; n=18 retinas for ECFCs-scrRNA EVs, n=20 retinas for ECFCs-shCD44 EVs, n=16 retinas for PBS, n=14 retinas for untreated. Error bars represent SEM.

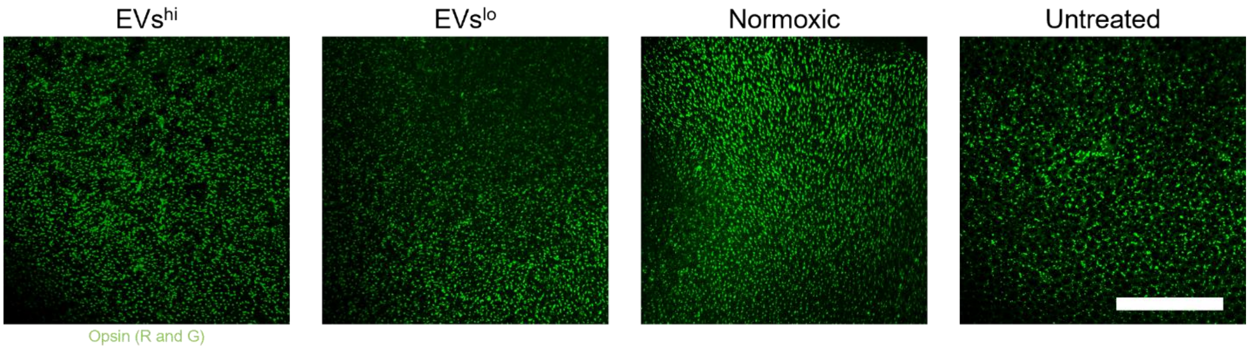

Supplemental Figure 7: **EVs^{hi} improved cone photoreceptor density in inherited retinal degeneration mice.** Representative images of opsin red/green stained retinal flat-mounts of P28 RD10 mice treated on P14 with EVs^{hi}, EVs^{lo}, or untreated controls versus normoxic BL6 mice.

miRs downregulated in EVs^{hi}	miRs upregulated in EVs^{hi}								
	miR-7-5p	miR-30a-5p	miR-503-5p	miR-221-3p	miR-23a-3p	miR-100-5p	miR-381-3p	miR-216a-3p	miR-181b-5p
miR-409-3p	0.80	-7.70	5.08	-0.86	1.92	5.40	-5.24	12.37	2.14
miR-30d-5p	7.49	-0.75	11.87	5.92	8.42	12.19	2.10	18.87	8.93
miR-191-5p	6.38	-1.92	10.67	4.72	7.68	10.99	0.15	18.13	7.73
miR-26a-5p	5.11	-3.13	9.67	3.66	6.66	9.82	-0.69	18.62	6.65
miR-584-5p	1.34	-6.05	5.83	-0.12	3.01	6.15	-4.52	13.46	2.89
miR-26b-5p	4.47	-4.12	8.74	2.79	5.68	9.06	-1.72	16.13	5.80
miR-671-3p	23.44	-6.81	22.82	10.32	15.31	17.50	-15.19	37.88	13.89
miR-335-3p	4.63	-4.21	9.34	3.08	10.10	9.00	-4.10	25.10	6.20
miR-486-5p	3.50	-4.64	8.03	2.10	5.40	8.33	-0.90	15.85	5.08
miR-128-3p	8.83	1.03	13.17	7.23	10.29	13.49	2.73	20.73	10.33

Supplemental Table 1: RT-qPCR validation of differentially expressed miRs on small RNA sequencing.

Reagent	Company	Host species	Catalog No.	Dilution Factor
**Flow cytometry on ECFCs**				
CD44 conjugated to APC	BD PharMingen	Mouse	559942	5 µL / test (1 x 10*6 cells in 100 µL buffer)
CD13 conjugated to APC	BioLegend	Mouse	301705	5 µL / test (1 x 10*6 cells in 100 µL buffer)
CD14 conjugated to FITC	BD PharMingen	Mouse	557153	5 µL / test (1 x 10*6 cells in 100 µL buffer)
CD31 conjugated to FITC	BD PharMingen	Mouse	560984	5 µL / test (1 x 10*6 cells in 100 µL buffer)
CD45 conjugated to FITC	BD PharMingen	Mouse	560976	5 µL / test (1 x 10*6 cells in 100 µL buffer)
CD90 conjugated to PE	BD PharMingen	Mouse	561970	5 µL / test (1 x 10*6 cells in 100 µL buffer)
CD105 conjugated to APC	BD PharMingen	Mouse	562408	5 µL / test (1 x 10*6 cells in 100 µL buffer)
VEGFR-2 conjugated to PE	R & D Systems	Mouse	FAB357P	5 µL / test (1 x 10*6 cells in 100 µL buffer)
HLA-A,B,C conjugated to PE	BioLegend	Mouse	311405	5 µL / test (1 x 10*6 cells in 100 µL buffer)
HLA-DR conjugated to APC	BioLegend	Mouse	307609	5 µL / test (1 x 10*6 cells in 100 µL buffer)
**Flow cytometry on EVs**				
CD9-Exo-Flow Capture Kit	SBI		EXOFLOW100A-1	10 µL capture antibody / 100 µL beads / 10 µL detection antibody / test
CD31-Exo-Flow Capture Kit	SBI		EXOFLOW200A-1	10 µL capture antibody / 100 µL beads / 10 µL detection antibody / test
CD63-Exo-Flow Capture Kit	SBI		EXOFLOW300A-1	10 µL capture antibody / 100 µL beads / 10 µL detection antibody / test
CD81-Exo-Flow Capture Kit	SBI		EXOFLOW400A-1	10 µL capture antibody / 100 µL beads / 10 µL detection antibody / test
**Immunohistochemistry**				
PECAM-1	BD PharMingen	Rat	553370	1:200
MAP2	Sigma	Mouse	M4403	1:1000
Arrestin	EMD Millipore	Rabbit	AB15282	1:200
Rhodopsin	EMD Millipore	Mouse	MABN15	1:1000
Opsin (Red/Green)	EMD Millipore	Rabbit	AB5405	1:200
Isolectin GS IB-4	ThermoFisher	N/A	I21413	1:200
Iba1	Wako	Rabbit	019-19741	1:200

Supplemental Table 2: **Reagents for flow cytometry and immunohistochemical staining.**

Product Line	Target	Company	Assay ID
**Transduction efficiency measurements**			
TaqMan Gene Expression Assay	CD44	ThermoFisher	Hs00229023_m1
TaqMan Gene Expression Assay	DICER1	ThermoFisher	Hs01075862_m1
TaqMan Gene Expression Assay	GAPDH	ThermoFisher	Hs02758991_g1s
TaqMan MicroRNA Assay	hsa-miR-7-5p	ThermoFisher	005723_mat
TaqMan MicroRNA Assay	hsa-miR-216a-3p	ThermoFisher	475580_mat
TaqMan MicroRNA Assay	hsa-miR-503-5p	ThermoFisher	001048
TaqMan MicroRNA Assay	U6 snRNA	ThermoFisher	001973
**RT-qPCR validation of small RNA sequencing results**			
TaqMan Advanced miRNA Assay	hsa-miR-7-5p	ThermoFisher	483061_mir
TaqMan Advanced miRNA Assay	hsa-miR-23a-3p	ThermoFisher	478532_mir
Taqman Advanced miRNA Assay	hsa-miR-26a-5p	ThermoFisher	477995_mir
Taqman Advanced miRNA Assay	has-miR-26b-5p	ThermoFisher	478418_mir
TaqMan Advanced miRNA Assay	hsa-miR-30a-5p	ThermoFisher	479448_mir
Taqman Advanced miRNA Assay	hsa-miR-30d-5p	ThermoFisher	478606_mir
TaqMan Advanced miRNA Assay	hsa-miR-100-5p	ThermoFisher	478224_mir
Taqman Advanced miRNA Assay	hsa-miR-128-3p	ThermoFisher	477892_mir
TaqMan Advanced miRNA Assay	hsa-miR-181b-5p	ThermoFisher	478583_mir
Taqman Advanced miRNA Assay	hsa-miR-191-5p	ThermoFisher	477952_mir
TaqMan Advanced miRNA Assay	hsa-miR-216a-3p	ThermoFisher	478770_mir
TaqMan Advanced miRNA Assay	hsa-miR-221-3p	ThermoFisher	477981_mir
Taqman Advanced miRNA Assay	hsa-miR-335-3p	ThermoFisher	478033_mir
TaqMan Advanced miRNA Assay	has-miR-381-3p	ThermoFisher	477816_mir
Taqman Advanced miRNA Assay	hsa-miR-409-3p	ThermoFisher	478084_mir
Taqman Advanced miRNA Assay	hsa-miR-486-5p	ThermoFisher	478128_mir
TaqMan Advanced miRNA Assay	hsa-miR-503-5p	ThermoFisher	478143_mir
Taqman Advanced miRNA Assay	hsa-miR-584-5p	ThermoFisher	478167_mir
Taqman Advanced miRNA Assay	has-miR-671-3p	ThermoFisher	478194_mir

Supplemental Table 3: Reagents for RT-qPCR.
